# Analysis of Genome Structure and Its Variations in Potato Cultivars Grown in Russia

**DOI:** 10.3390/ijms24065713

**Published:** 2023-03-16

**Authors:** Dmitry I. Karetnikov, Gennady V. Vasiliev, Stepan V. Toshchakov, Nikolay A. Shmakov, Mikhail A. Genaev, Mikhail A. Nesterov, Salmaz M. Ibragimova, Daniil A. Rybakov, Tatjana A. Gavrilenko, Elena A. Salina, Maxim V. Patrushev, Alex V. Kochetov, Dmitry A. Afonnikov

**Affiliations:** 1 Federal Research Center Institute of Cytology and Genetics SB RAS, 630090 Novosibirsk, Russia; 2 Kurchatov Center for Genome Research, Institute of Cytology and Genetics SB RAS, 630090 Novosibirsk, Russia; 3 Faculty of Natural Sciences, Novosibirsk State University, 630090 Novosibirsk, Russia; 4 Kurchatov Center for Genome Research, National Research Center Kurchatov Institute, 123182 Moscow, Russia; 5 Federal Research Center the N.I. Vavilov All-Russian Institute of Plant Genetic Resources (VIR), 190000 St. Petersburg, Russia; 6 Faculty of Agronomy, Novosibirsk State Agrarian University, 630039 Novosibirsk, Russia

**Keywords:** potato, *Solanum tuberosum* L., breeding cultivars, genome structural variation, copy number variation, potato diversification, genome annotation, NBS-LRR genes, photoperiod control, phytochrome A, poly(ADP-ribose) glycohydrolase

## Abstract

*Solanum tuberosum* L. (common potato) is one of the most important crops produced almost all over the world. Genomic sequences of potato opens the way for studying the molecular variations related to diversification. We performed a reconstruction of genomic sequences for 15 tetraploid potato cultivars grown in Russia using short reads. Protein-coding genes were identified; conserved and variable parts of pan-genome and the repertoire of the NBS-LRR genes were characterized. For comparison, we used additional genomic sequences for twelve South American potato accessions, performed analysis of genetic diversity, and identified the copy number variations (CNVs) in two these groups of potato. Genomes of Russian potato cultivars were more homogeneous by CNV characteristics and have smaller maximum deletion size in comparison with South American ones. Genes with different CNV occurrences in two these groups of potato accessions were identified. We revealed genes of immune/abiotic stress response, transport and five genes related to tuberization and photoperiod control among them. Four genes related to tuberization and photoperiod were investigated in potatoes previously (phytochrome A among them). A novel gene, homologous to the poly(ADP-ribose) glycohydrolase (PARG) of *Arabidopsis*, was identified that may be involved in circadian rhythm control and contribute to the acclimatization processes of Russian potato cultivars.

## 1. Introduction

Common potato (*Solanum tuberosum* L.) is one the most important crops grown worldwide. It is ranked the first highest produced non-cereal food crop and the fourth highest produced crop worldwide after wheat, corn, and rice [1]. Potato is produced as food and animal feed, and they are also grown for industrial purposes. Potatoes contribute key nutrients to the human diet including vitamin C, potassium, and dietary fiber [2].

The latest taxonomical treatment of Spooner et al. [3,4] recognized four cultivated potato species—*S. tuberosum*, divided into Chilean and Andean cultivar groups, and three hybrid cultivated species of “bitter potato”, *S. ajanhuiri* Juz. and Bukasov, *S. curtilobum* Juz. and Bukasov, *S. juzepczukii* Buk.). Thus, *S. tuberosum* is represented by native tetraploid cultivars (landraces) grown in the lowlands of Chile (*S. tuberosum* Chilotanum group) and by populations of di-, tri-, and tetraploid landraces grown in the highlands of the Andes (*S. tuberosum* Andigenum group). Continuing breeding has led to the development of thousands of improved cultivars; in most cases, modern improved cultivars are the product of interspecific crosses with different cultivated and wild potato species [4,5]. However, there is still a need worldwide to develop new cultivars with desirable and more effective properties.

Modern potato cultivars are autotetraploid with tetrasomic inheritance, high heterozygosity, frequent pollen sterility, and clonal method of reproduction [6]. The latter factor causes dysfunctional and deleterious alleles not to be removed during meiosis, leading to inbred depression. These factors cause complications in potato breeding [7]. In this regard, genome sequencing and analysis provide the basis for efficient research in the field of potato genetics and breeding.

The potato genome of the homozygous DM1-3 double monoploid was sequenced and assembled by the Potato Genome Sequencing Consortium [8]. Subsequently, additional DM1-3 pseudomolecules with improved sequence and annotation quality were assembled and annotated [9,10,11]. Extensive CVNs were found, affecting more than 30% of the potato genome [11]. Lately, a high-quality haplotype-resolved assembly to the chromosomes of the diploid [12,13] and autotetraploid potato cultivar (cv.) *S. tuberosum* “Otava” genome [14] was obtained using long reads and the chromosome conformation capture method. Recently the phased *S. tuberosum* genomes of several commercial cultivars from North America and Europe were sequenced and assembled [15]. Using long reads, an assembly was obtained for high-quality diploid potato genomes from more than 40 wild and cultivated representatives of the *Solanum* section *Petota* (including diploid Andean landraces) [6]. Phased potato genome assemblies allow the characterization of the potato pan-genome, the study of global rearrangements at the chromosome level between haplotypes and opens up new possibilities for directed breeding in the future.

In addition to high quality assemblies based on long reads, methods of genomic sequence reconstruction based on short reads are actively used. Recently, genomic sequences for six accessions of cultivated potato polyploid species were assembled, three of which were tetraploid landraces belonging to the Andigenum group and to the Chilotanum group and two of them were sequenced only by short reads [16]. Nucleotide and structural variations were analyzed by short-read sequencing in various potato species and landraces from South America [17], elite tetraploid cultivars [18], potato somatic hybrid, its parents and progeny [19], diploid potato clones [20]. These data made it possible to estimate such structural characteristics of the potato genomes as the set of protein-coding genes [16,19], characterize their functions [16], estimate copy number variations, single nucleotide, and small insertion–deletion polymorphisms [17,18,19,20].

Copy number variations play an important role in crop domestication and diversification [21]. In potato genomes, they provide a major contribution to the genomic diversity of clonally propagated potatoes, as well as affecting species-specific and dispensable groups of pan-genome genes [11]. Using identified CNVs, it is possible to analyze their contribution to the genetic diversity of wild and cultivated species accessions, as well as identify clusters of genes that are affected by CNVs in potato genomes (such as SAUR, gene clusters, gene clusters of metabolite biosynthesis, etc.) [17].

The aim of our work is to study the diversity and variation of genomic sequences of potato cultivars grown in Russia and to search for genes that could participate in their diversification and acclimatization through copy number changes based on CNV comparison for Russian potato cultivars and South American accessions sequenced recently. We reconstructed genome sequences using short read sequencing for 15 potato-improved cultivars, 14 developed by different Russian breeding programs, and 1 Dutch variety cultivated in Russia (further, we will call this subset Russian cultivars). We identified the conserved and variable parts of the pan-genome and estimated the functions associated with them, assessing the diversity of NBS-LRR genes. Using additional sequence data of 12 genomes of South American potato species accessions represented by 1 wild ancestor species *S. bukasovii* and 11 accessions of Andean and Chilean cultivated species, we performed a comparative analysis of CNVs, which allowed us to identify structural gene variations with different occurrence in these 2 subsets. Functional analysis of these genes was performed, and it was shown that they are associated with the response to abiotic and biotic stresses. A number of genes from this pool have been identified, which are associated with tuberization and control of circadian rhythms.

## 2. Results

### 2.1. Genome Assembly Statistics and Annotation for Russian Cultivars

The number of reads in our libraries varies from 60 million to 290 million, yielding the sequence coverage of the *S. tuberosum* group phureja DM1-3 reference potato genome from 23× for cv. Udacha to 109× for cv. Grand (Table S1, Supplementary File S1). The proportion of paired-end reads for all cultivars after preprocessing was more than 87% (Table S1, Supplementary File S1).

Table 1 shows the main statistics for the genomes of Russian potato cultivars. The number of contigs ranged from 196 kb (cv. Grand) to 551 kb (cv. Udacha), and the proportion of contigs smaller than 1000 bp without an open reading frame was from 35% (cv. Udacha) to 80% (cv. Fritella). The total lengths of assembled and filtered contigs exceeded the size of the DM1-3 reference genome, 810 Mb for all but one accession (cv. Udacha, 653 Mb) (Table 1). The GC content of the genomic sequences varies from 34.83% (cv. Nikulinsky) to 36.22% (cv. Gusar), the average for all genomes being 35.55% (34.84% for DM1-3). The contigs of largest length range from 163 Mb (cv. Krasa Meshchery) to 281 Mb (cv. Krepysh); N50 varies from 8095 bp (cv. Gusar) to 15,505 bp (cv. Grand) and shows a positive relationship with the coverage value. The average proportion of identified repeats is 61%. The statistics (Table 1) indicate the fragmentation of our assemblies is high.

The results of BUSCO analysis (Figure S1, Supplementary File S1) demonstrated that of Solanales dataset proteins more than 60% are present in all genomes of Russian potato cultivars completely and in a single copy. The fraction of duplicated proteins varies from 2.5% for cv. Udacha to 13% for cv. Fritella. The fraction of fragmented variants vary from 6% for cv. Grand to 12% for cv. Udacha. The fraction of missed proteins varies from 15% for cv. Zhukovsky to 24% for cv. Gusar.

### 2.2. Protein Orthogroups Analysis

The results of the identification of orthologous groups for protein-coding genes for 15 Russian potato cultivars, and 12 South American accessions from ref. [17], DM1-3 reference genome and the tomato *Solanum lycopersicum* genome (outgroup) are presented in Table 2. The number of open reading frames (ORFs) for Russian cultivars varies from 60,411 (cv. Udacha) to 77,417 (cv. Krasa Meshchery), this value is higher for genomes with high coverage (for DM1-3 39,021 ORFs are known). More than 90% of the proteins in all South American accessions, Russian cultivars, and reference genomes belong to common orthologous groups.

The total number of orthologous groups identified is 125,744, of which 84,450 groups have more than 1 sequence and include 2,117,217 sequences. The remaining 41,294 amino acid sequences (2% of the total number of sequences and 33% of the total number of orthogroups) represent unassigned (single-sequence) orthogroups. The average number of sequences in the orthologous group is 25.1, with a median of 9.0. The G50 metric (the number of genes in the orthogroup such that 50% of genes are in orthogroups of that size or larger) is 56 sequences, and the O50 metric (the smallest number of orthogroups such that 50% of genes are in orthogroups of that size or larger) is 9220. Only seven orthologous groups include exactly one sequence from each genome.

### 2.3. Analysis of ORFs in Russian Cultivars

**Figure 1:**
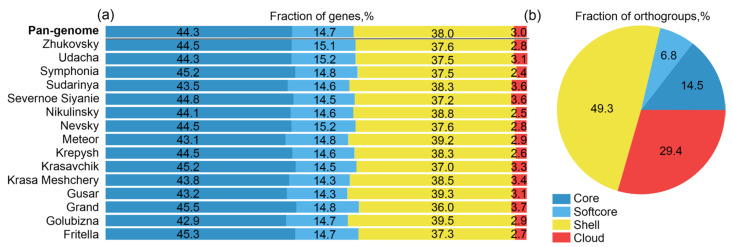
Summary assessments for the proportion of genes (**a**) and orthogroups (**a**) present in pan-genome of 15 assemblies of Russian potato cultivars. (**a**) Bar chart for the proportion of core, softcore, shell, and cloud genes in the pan-genome (top bar) and each cultivar. *X*-axis: the fraction of genes. *Y*-axis: cultivars denoted by abbreviations. (**b**) Pie chart for the distribution of core, softcore, shell, and cloud orthogroups in the pan-genome. Color legend for pan-genome parts is shown below the pie chart.

In the group of 15 genomes of potato cultivars grown in Russia, 103,748 orthologous groups were identified. Genes in these orthogroups were classified into “core”, “softcore”, “shell” and “cloud” types by their occurrence in different accessions (see Methods, Section 4.6). The fraction of genes of these types in pan-genome and each genome is shown in Figure 1a. Of the 1,050,536 genes annotated, 465,278 (44.5% of the total number) belong to the “core” part; for individual cultivars, the proportion of this type of gene varies from 42% to 45.5%. The number of genes belonging to “shell” orthogroups is 399,045 genes (37.6%). The number of genes belonging to “softcore” orthogroups is 154,283 (15.1%) and the number of genes belonging to “cloud” orthogroups is 31,930 (2.8%). According to the ratio of genes in these categories of orthogroups, the Russian cultivars are similar, despite the difference in genomic sequence coverage by reads.

The distribution of orthogroups by the represented genome number is shown in Figure 1b. Orthogroups that contain genes from 2 to 13 genomes (“shell” genes) prevail (51,138 orthogroups). The second largest category is orthogroups represented in a single genome (“cloud”, 30,516 orthogroups). Next, are 14,992 orthogroups containing genes in all 15 genomes (“core”), and 7102 orthogroups represented in at least 14 genomes (“softcore”). The difference between fractions of “core”, “softcore”, “shell”, and “cloud” parts for genes (Figure 1a) and orthogroups (Figure 1b) can be explained by several homologs of genes in one orthogroup representing the same genome.

**Figure 2:**
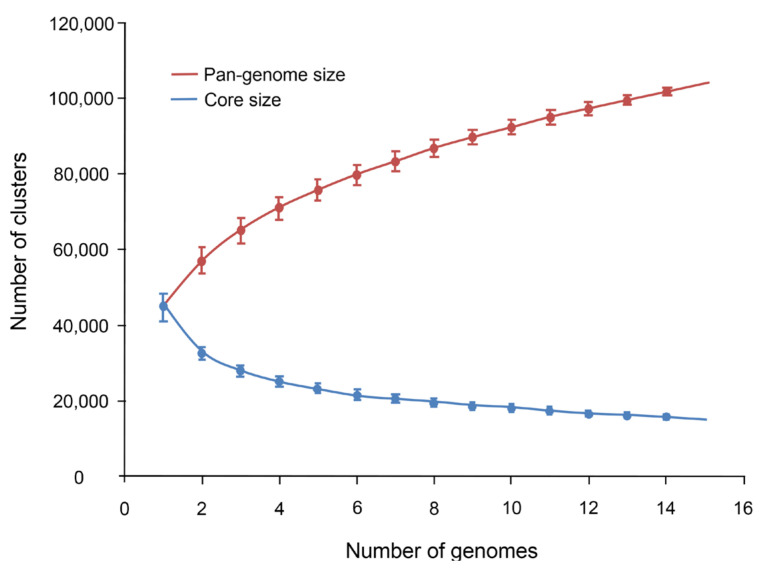
Simulation of pan- and core-genome sizes, in terms of number of orthologous gene clusters and pan-genome composition. *X*-axis is the number of genomes; the *Y*-axis is the number of clusters. The plot color legend is provided for each part.

Using the data on the number of genes in the orthologous groups, we estimated the change in the size of the pan-genome of Russian potato cultivars and its conserved part (Figure 2). The plot demonstrates that the size of the potato pan-genome does not reach the plateau for the number of genomes we investigated.

We performed functional annotation of protein-coding genes in Russian cultivars. The number and percentage of annotated ORFs are shown in Table 3: the proportion of annotated sequences for all cultivars exceeds 53%, and the maximum fraction of annotated genes is observed for cv. Grand (59.04%), the minimum is observed for cv. Gusar (53.18%).

The proportion of annotated genes in the “core” part of the pan-genome (66.1%) is only slightly higher than in the “softcore” (61.5%), one and a half times higher than in the “shell” (41.4%), and almost three times higher than in the “cloud” (22.5%). We identified the 15 most frequent functional domains in the pan-genome and estimated their frequencies in different parts of the pan-genome. The results are shown in Figure S2 (Supplementary File S1) and indicate that domains such as cytochrome P450, protein kinases and tyrosine kinases, RNA-recognition motif, F-box, and PPR repeats have a higher frequency of occurrence in the conserved part of the pan-genome (“core”). Such domains as NB-ARCs, LRR motifs, integrases, gag domains, and reverse transcriptases are characterized by higher frequency in the variable part of the pan-genome (“cloud”, “shell”).

### 2.4. Analysis of NBS-LRR Genes

We identified 3404 full-length proteins of the NBS-LRR family in the amino acid sequences of Russian potato cultivars. Based on the co-occurrence in the orthogroups with known NLR-proteins of the reference genome DM1-3, NBS-LRR classes were identified for 1270 sequences. The remaining 2134 proteins fall into orthogroups with no NLR-proteins from the reference DM1-3 and thus were not attributed to particular NLR classes. Table 4 shows the number of NLR genes of each class predicted in the genome of an individual cultivar. The proportion of proteins of different classes among all NBS-LRR proteins is shown in Figure S3 (Supplementary File S1).

The highest number of NBS-LRR proteins (306) was identified for the cv. Severnoe Siyanie, the lowest (135), for the cv. Udacha (Table 4). The number of NBS-LRR correlates well with the coverage of the genome by reads (the higher the coverage, the more proteins identified). Four cultivars with the highest number of NBS-LRR genes (from 281 to 306) have coverage greater than 100× (cvs. Krasavchik, Grand, Fritella, Severnoe Siyanie). Four cultivars with the lowest number of NBS-LRR genes, from 135 to 182 (cvs. Udacha, Sudarinya, Zhukovsky, Gusar), have low coverage (23× to 48×). On average, there are 227 NLR proteins per genome. The most represented classes of the NBS-LRR proteins are CNL-1 (267 proteins in all 15 genomes), CNL-7 (251 proteins), and CNL-R (198 proteins); the least represented class is CNL-4, which contains a total of 11 genes in all 15 genomes.

Our data indicate a high diversity of NBS-LRR genes in the 15 Russian potato cultivars we studied. This is evidenced by the proportion of proteins that were assigned to orthogroups that did not include proteins from the DM1-3 genome (60% and higher) (Figure S3, Supplementary File S1). Clearly, they represent the variable part of the immune response proteins.

### 2.5. Genetic Differentiation among Improved Russian Cultivars and South American Landrace Accessions

**Figure 3:**
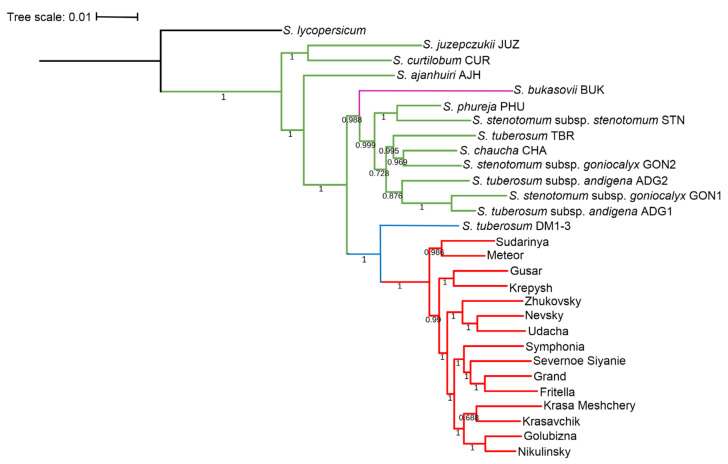
Genetic differentiation within two subsets of South American landraces and Russian potato cultivars accessions, as well as the reference tomato and potato DM1-3 genomes, built by Orthofinder program. Branches leading to Russian cultivars are shown in red, to South American cultivated species accessions—in green. Wild species *S. bukasivii* (BUK) is shown by magenta branch. The branch corresponding to the reference genome (DM1-3 PGSM v4.03) is shown in blue, the S. lycopersicum outgroup is shown in black. The scale bar is shown in the upper left corner, the numbers at the nodes correspond to the Shimodaira–Hasegawa-like support values.

To study genetic differentiation and genome diversity of cultivated potatoes (Table 2), 1012 orthologous groups were used by OrthoFinder, in which at least 51.7% of amino acid sequences represented a single copy for any of the genotypes. The resulting tree of genetic differentiation is shown in Figure 3.

One can see from Figure 3 that Russian potato cultivars form a cluster (support value 1) whose members are separated from the studied South American landraces. This cluster separates into several clusters of smaller sizes. The level of support for the cluster of South American landrace genotypes (*S. tuberosum* Andigenum group and *S. tuberosum* Chilotanum group) is also high (0.999). At the root of the cluster is the wild ancestor species *S. bukasovii* Juz. (=*Solanum candolleanum* Berthault). The remaining accessions of three highlands “bitter” cultivated potato species (*S. juzepczukii*, *S. curtilobum*, and *S. ahanhuiri*) form the group at the root of all other potato genotypes. These three species are of hybrid origin derived from natural crosses between wild species *S. acaule*, *S. boliviense* and cultivated species distributed in the high Andean altiplano between Southern Peru and Central Bolivia, at elevations between 3600 and 4400 m [22,23]. Thus, characteristic of the tree is the location of three “bitter” potato species from Andean highlands at the root of the tree and the presence of two sister groups of Russian cultivars and nine South American potato accessions.

### 2.6. CNV Characteristics for Russian Cultivars and South American Landrace Potato Accessions

**Figure 4:**
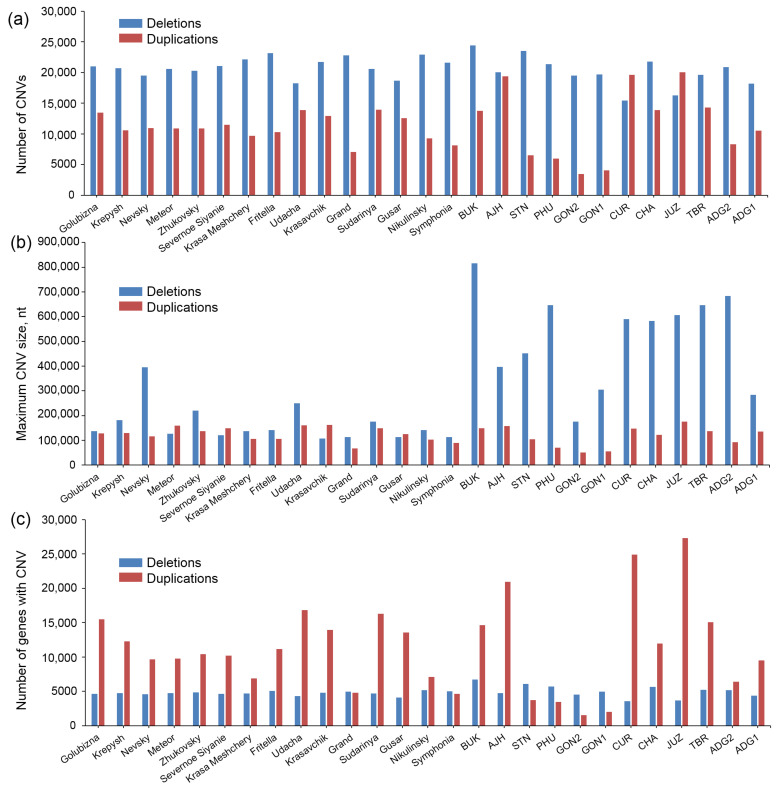
Distribution of CNV quantitative characteristics in the genomes of Russian cultivars and South American potato species accessions. (**a**) Distribution of the number of duplications and deletions; (**b**) distribution of maximum lengths of deletions and duplications; (**c**) distribution of the number of genes with deletions and duplications. See Table 2 for abbreviation of the names of South American accessions.

Bioinformatics analysis using CNVpytor v0.4.1 allowed CNV identification and analysis in Russian potato cultivars, and South American landrace potato accessions and compare their characteristics. The distribution of CNVs for different accessions by number, maximal length, and related gene numbers is shown in Figure 4 and Table S2 (Supplementary File S1).

Figure 4a shows that the number of deletions for most genotypes exceeds the number of duplications (except for South American CUR and JUZ accessions). Russian cultivars compared to South American ones are more homogeneous in this parameter: the number of duplications varies near 10,000 except for the cv. Grand. The number of deletions varies by about 20,000 without significant deviations for any cultivars. At the same time, sharp differences in the number of duplications are observed for the South American samples: their number is ~5000 or less for STN, PHU, GON2, and GON1; high values of the number of duplications (more than 14,000) for BUK, AJH, CUR, and JUZ; moderate values, comparable to those of Russian cultivars for CHA, TBR, ADG2, and ADG1. The number of deletions approximately corresponds to that in Russian cultivars, except for the aforementioned CUR and JUZ (the lowest values among all genotypes). Interestingly, the CUR, JUZ, and AJH accessions (the number of deletions is greater than the number of duplications or close to it) belong to the group of highland “bitter” cultivated potato species located at the root of tree in Figure 3.

The distribution of the maximum size of the duplicated or deleted segment demonstrates a high homogeneity of Russian cultivars and diversity of South American accessions (Figure 4b). The maximum lengths of deletions/duplications for Russian cultivars are close to ~100,000 bp, for some cultivars the maximum length of deletions is slightly greater than the number of duplications and for others vice versa. The length of structural rearrangements is somewhat less in the cv. Grand, and cv. Nevsky is characterized by a significant excess of the maximum length of deletions (400,000 bp) over the maximum length of duplications (~100,000 bp). The Russian cultivars also demonstrate uniformity in the average size of rearrangements, close to 9000 bp except for the mentioned cv. Grand (value ~6500 bp).

A number of South American accessions are characterized by a significantly longer length of the deleted segment compared to the duplicated one. Thus, for BUK wild species accession it is over 800,000 bp, while for PHU, CUR, CHA, JUZ, TBR, and ADG2 accessions it is over 500,000 bp. At the same time, for the GON2 genotype, this value is comparable to that of Russian cultivars, while for GON1 and ADG2 it is only two times higher. The maximum length of duplications for the South American genotypes does not differ significantly from the Russian ones (not more than 2 times, maximum 175,000 bp for JUZ), but compared to the Russian cultivars, the variability of South American landraces in this parameter is higher.

While the number of deletions in the genomes we studied is in most cases higher than duplications, they affect a smaller number of protein-coding genes (ORFs) compared to duplications (Figure 4c). Interestingly, the genotypes we studied are rather homogeneous in the number of genes affected by deletions: there are no remarkable differences between the Russian and South American accessions. In terms of the number of genes affected by duplications, on the contrary, the variability is high, also among Russian cultivars. The low number of duplications (less than 5000 bp) affects genes in cvs. Grand, Symphonia, and diploid cultivated species accessions from South America—STN, PHU, GON2, GON1. A high value is observed in both Russian cultivars (Golubizna, Udacha, Krasavchik, Sudarynia, Gusar) and South American accessions (BUK, AJH, CUR, JUZ, TBR). The remaining accessions show a moderate number of gene duplications.

We performed statistical tests for a difference in means and the equality of two variances for CNV characteristics from Figure 4 in Russian cultivars and South American accessions (Table S3, Supplementary File S1). The results demonstrated that the mean values differ significantly for one CNV characteristic (maximum deletion size, Figure 4b). Variances are unequal for all characteristics except maximum duplication size (Figure 4b). These results support the hypothesis that Russian cultivars compared to South American ones are more homogeneous in most CNV parameters.

The distribution of CNV on chromosomes of both accessions from Russia and South America is shown in Figures S4 and S5 (Supplementary File S1). These figures demonstrate that CNVs are distributed unevenly both along the genome and between accessions and that CNV distribution patterns on chromosomes in Russian and South American cultivars differ. For example, for chromosome 1, all studied cultivars are characterized by a low number of duplications at the 25-30 Mb region (white pattern in Figure S4). At the same time, a number of Russian cultivars are characterized by the less frequent occurrence of CNV at 65-90 Mb (lighter shade): cv. Krasa Meshchery, cv. Grand, cv. Symphonia, cv. Nikulinsky. The same differences are observed for these cultivars on the second chromosome, and in general, they show fewer duplications compared to the remaining cultivars from Russia (in agreement with Figure 4a). Diploid Andean landraces of STN, PHU, GON2, and GON1 show similar differences relative to the rest of the South American accessions. The density of CNV duplications for them is lower on almost all chromosomes than for the rest of the South American polyploid landraces. This is also consistent with the data in Figure 4a.

A number of the chromosome segments corresponding to the differences between Russian and South American accessions are noticeable. For example, the duplications on chromosome 7: Russian cultivars are depleted in them around 25 Mb, while for South American landraces this is not observed (Figure S4, Supplementary File S1). The lower density of duplications is also characteristic of Russian cultivars in the central parts of chromosomes 11 and 12.

Figure S5 (Supplementary File S1) demonstrates no clearly distinguishable accessions that were enriched with deletions in either Russian or South American accessions (which is also consistent with Figure 4a). At the same time, a number of genome segments are noticeable in which there are differences between Russian and South American accessions. For example, the frequency of deletions in Russian cultivars is lower than in South American accessions in chromosome 10. This is also characteristic of chromosomes 3 and 9. In contrast, an increased frequency of deletions on chromosome 7 in a number of Russian cultivars is observed within the region of 0–30 Mb; a number of South American accessions are depleted of deletions in this region.

Thus, our analysis demonstrates the higher homogeneity of Russian cultivars in terms of genome structural rearrangements compared to South American cultivated potato accessions. These structural variations are characterized by the prevalence of deletions over duplications with respect to the whole genome and vice versa, duplications over deletions, in the regions containing protein-coding genes. In addition, there is great diversity in the distribution of CNVs on the chromosomes of the accessions we studied.

### 2.7. CNV-Based Similarity of Potato Genomes

We compared the potato genomes we studied by the similarity of CNV frequencies in the loci encoding proteins based on principal component analysis as proposed in [17]. The total number of DM1-3 v4.03 reference genome genes affected by CNV in 15 cultivars from Russia and 12 South American cultivars associated with CNV was 38,310.

**Figure 5:**
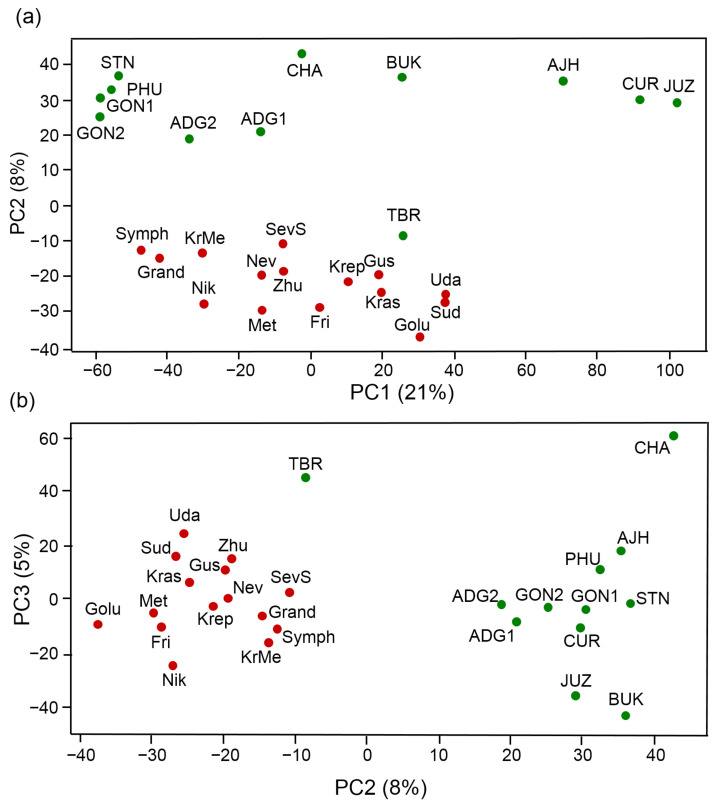
Principal component analysis diagrams for potato genotypes based on the CNV similarity for DM1-3 v4.03 protein-coding genes. (**a**) PCA plot for PC1 (*X*-axis) and PC2 (*Y*-axis) components; (**b**) PCA plot for PC2 (*X*-axis) and PC3 (*Y*-axis) components. Explained percentage of variance shown in parentheses. Selected for this study Russian potato cultivars shown as red dots, and South American accessions shown as green dots. See Table 1 for abbreviation of the names of 15 Russian cultivars and Table 2 for abbreviation of the names of South American accessions.

The principal component analysis shows that the first component accounts for 21%, the second 8%, and the third 5% of the total variance. The bivariate PCA diagrams for the projections to the first three components are shown in Figure 5. The diagram for the two principal components (Figure 5A) shows that the first component is characterized by the division of all samples into the species of hybrid origin from the high Andes (JUZ, CUR, AJH) and all others, with strong overlapping values for the rest of the genomes of the South American accessions and Russian cultivars in this component. Interestingly, the Andean diploid landraces STN, PHU, GON1, GON2 and Russian cvs. Symphonia, Grand, Nikulinsky, Krasa Meshchery have the lowest values of the first component and the lowest number of duplications (Figure 4a). At the same time, the highest values of this component are characteristic of South American allopolyploid cultivated species (CUR, JUZ) and diploid cultivated species *S. ahanhuiri* (AJH) having hybrid origin with the highest number of duplications (Figure 4a). Apparently, the first component in Figure 5a is likely related to the number of duplications.

The second component, on the contrary, is associated with the separation between genomes of analyzed Russian and South American potato accessions. It is interesting that in contrast to the tree in Figure 3 in the PC1/PC2 diagram Chilean *S. tuberosum* (TBR) is closer to the Russian cultivars than to South American Andean landraces. On the other hand, the PC2/PC3 diagram (Figure 5b) shows that this genotype is quite far from the Russian cultivars in the third component.

It should also be noted that the samples corresponding to the Russian cultivars in these graphs form a separate cloud. At the same time, among the South American accessions, several separate groups can be distinguished (Figure 5a). The first one mentioned above, JUZ, CUR, and AJH, corresponds to the “bitter” cultivated species accessions located at the root of the tree diagram of potato genotypes (Figure 3). The second one includes of Andean diploid landraces (GON2, GON1, PHU, STN); Andean cultivated tetraploid species (ADG1, ADG2) are located closer to them, all together they belong to the *S. tuberosum* Andigenum group. Accessions of Andean-cultivated triploid *S. chaucha* (CHA) and wild ancestor species *S. bukasovii* (BUK) are further away, and TBR is located close to Russian cultivars. Note that the location of accessions in the PCA diagram only at the level of large clusters corresponds to their genetic differentiation (Figure 3), while in the details (at closer distances) there are differences.

**Figure 6:**
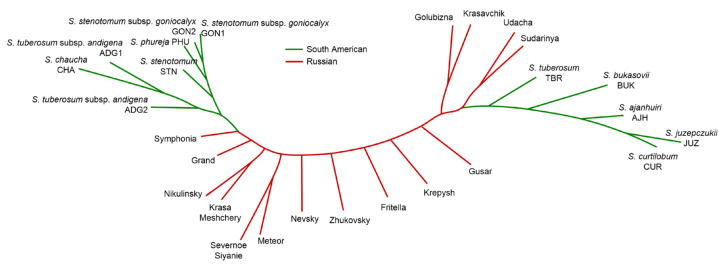
Tree diagram reconstructed by potato genomes using their CNV similarity. Cultivars from Russia are shown by red branch color and South American potato accessions shown by green branch color.

We also represented the similarity of analyzed potato genomes by CNV distribution in the form of the tree shown in Figure 6. Its structure is consistent with the diagrams obtained from the principal component analysis: the tree contains a cluster of AJH, JUZ, and CUR, which is joined by South American BUK and TBR genomes. The second cluster of genomes of South American accessions in this tree corresponds to the *S. tuberosum* Andigenum accessions located in the PCA1/PCA2 diagram in the upper left corner (Figure 5a): ADG1, ADG2, GON1, GON2, PHU, STN with CHA joining them. The Russian cultivars on this tree belong to mostly star-like branches from the main stem of the tree.

### 2.8. Comparison of the CNVs Occurrence in Genomes of South American Cultivated Species and Russian Cultivars

As noted above, the tree of genetic differentiation of potato genomes (Figure 3) is characterized by the presence of two sister clusters of Russian cultivars (including reference gene DM1-3) and South American cultivated species accessions (except for JUZ, CUR, and AJH). This allows us to search for loci that have different CNV occurrences in genomes from these two sister clusters of potatoes. We additionally excluded from the South American cluster the BUK genome, which represents a wild type and forms a long branch from the root of this cluster (shown in magenta in Figure 3). Therefore, we analyzed occurrences of CNVs related to protein-coding genes in genomes of 15 Russian cultivars and 8 South American cultivated species accessions.

Each CNV locus associated with a protein-coding gene in a particular genome was characterized by three CNV types according to our analysis: significant duplications (+1), significant deletions (-1), and without any significant CNV (0) (see Materials and Methods, Section 4.8). To identify loci in which occurrence of CNV of these types unevenly distributed between Russian and South American cultivars, we performed Fisher’s exact test (2 × 3 contingency table analysis). As a result, we identified 1742 genes for which this test showed significance at the *p*-value < 0.01 (Supplementary File S2).

We further narrowed the above list of genes by distinguishing specific patterns of CNV occurrence between genomes of studied Russian cultivars and South American accessions of several types (see Materials and Methods, Section 4.8): RUDup (duplications in Russian cultivars); SADup (duplications in South American landraces); RUDel (deletions in Russian cultivars); SADel (deletions in South American landraces). We identified 236 genes for RUDup, 33 for SADup, 109 for RUDel, and 219 for SADel CNV patterns (Supplementary File S2).

Enrichment analysis for the entire list of significant genes using the DAVID yielded 10 significant functional clusters (FDR < 0.05; Supplementary File S2). They represent the following terms: NB-ARC/plant defense/leucine-rich repeat (cluster 1); poly(ADP-ribose) glycohydrolase activity (cluster 2); replication factor A (cluster 3); vesicle-mediated transport/vacuolar sorting protein 39 (cluster 4); leucine-rich repeat (cluster 5); aspartic-type endopeptidase activity (cluster 6); voltage-gated anion channel activity/porin domain (cluster 7); FBD domain (cluster 8); RNA methylation (cluster 9); TIR/signal transduction (cluster 10).

No significant functional clusters were found for SADup and RUDel patterns (Supplementary File S2). For the group of 236 genes with the RUDup pattern, the following functional annotation clusters were identified: vesicle-mediated transport/vacuolar sorting protein 39 (cluster 1); RNA methylation (cluster 2); voltage-gated anion channel activity/porin (cluster 3); fucose metabolic process (cluster 4); GTPase activity/guanyl nucleotide binding (cluster 5); methyltransferase activity (cluster 6); isomerase/peptidyl-prolyl cis-trans isomerase activity (cluster 7); carbohydrate-binding/isomerase/aldose 1-epimerase activity (cluster 8); KDPG/KHG aldolase (cluster 9).

Four functional gene annotation clusters were found for the SADel pattern (Supplementary File S2): polyphenol oxidase (cluster 1); ascorbate biosynthesis/inositol oxygenase activity (cluster 2); peptidase M76, ATP23 (cluster 3); translation elongation factor EF1A (cluster 4).

### 2.9. Comparison of the CNVs Occurrence in Genomes of South American and Russian Potato Accessions

Genes associated with tuberization and photoperiod control in potatoes are actively studied in their relationship to adaptation to the long day typical for European latitudes [4,24]. To evaluate the influence of CNV on these genes in genomes of Russian and South American accessions, we searched for genes associated with tuberization and photoperiod control (Supplementary File S3) among the genes with significant differences in CNV occurrence (Supplementary File S2). We found such differences for four genes (Table 5).

In addition to the genes identified previously as associated with tuberization and photoperiod control in potatoes (Supplementary File S3), we found another gene, Poly(ADP-ribose) glycohydrolase, which demonstrated different CNV occurrence in genomes of Russian and South American accessions analyzed in the present study (Table 5). We identified significant duplications of this gene for all 15 Russian cultivars, and only for 3 South American accessions (CHA, TBR, ADG2). The homolog of this gene in *Arabidopsis*, PARG, is involved in circadian rhythm control [27].

According to Table 5, deletions are common for tuberization and photoperiod control genes in South American potato accessions (four genes out of five). In Russian cultivars, a remarkable number of deletions, 4 accessions out of fifteen, is observed only for gene PGSC0003DMG400012838, while in South American cultivars the number of accessions with deletions for this gene is seven out of eight. Other genes in Table 5 are characterized by the absence of any frequent deletions in Russian genomes and by the remarkable proportion of duplications (8 of 15 for the gene PGSC0003DMG400015766 and 15 of 15 for the gene PGSC0003DMG400029361).

### 2.10. CNVs in SAUR Gene Clusters

Previously, CNV analysis in potatoes demonstrated the presence of a large number of structural variations in the loci associated with SAUR (small auxin-up RNA) gene clusters involved in auxin signaling [11]. These are gene clusters located on chromosomes 1 (86.97-87.17 Mb), 4 (54.17-54.37 Mb), 6 (56.29-56.49 Mb), and 11 (0.87-1.11 Mb) [17]. Our analysis showed that these loci are enriched in CNV not only in South American landraces but also in Russian cultivars (Figures S6–S9, Supplementary File S1). The cluster of SAUR genes on chromosome 1 demonstrates the greatest CNV enrichment (Figure S6, Supplementary File S1). On chromosome 4, the dominance of duplications in Russian cultivars over deletions and over CNV of both types in South American accessions (Figure S7, Supplementary File S1) was observed. However, we observed significant differences in CNV occurrence in the South American and Russian accessions only for two SAUR genes. The first gene PGSC0003DMG400016568 (auxin-induced SAUR) located on chromosome 4 at ~54.187 Mb (Supplementary File S2, see also Figure S7, Supplementary File S1) and affected by deletions in four South American potato genomes and is not affected by any CNV in Russian genomes. The second gene is PGSC0003DMG400021565 (SAUR family protein), for which 10 duplications in Russian cultivars and no significant CNVs in South American potato accessions are observed (Supplementary File S2). Interestingly, this gene does not belong to any of the mentioned clusters and is located on chromosome 9 at position ~54.81 Mb. Thus, despite the presence of a large number of CNVs in the SAUR cluster regions, their specificity with respect to Russian or South American accessions is weakly expressed.

## 3. Discussion

### 3.1. Genome Assemblies

We used short-read sequencing to analyze the genomic sequences of 15 potato cultivars grown in Russia. Analysis of the quality of their genome assemblies showed that the N50 values for our assemblies are slightly higher than that obtained in assemblies of South American potato accessions based on Illumina sequencing [16]. L50 values in our assemblies are lower than for genomes of South American accessions, also indicating the generally longer contigs that we were able to assemble. Given higher coverage for a number of genomes of Russian cultivars, the results can be considered generally comparable. In terms of the number of identified ORFs, our results are also consistent with the analysis of the South American accessions [16]. We identified about 80% of the genes from the BUSCO proteins (approximately the same proportion for all accessions), which is also comparable to the results for South American polyploid landraces [16]. Note that for the four haplotypes of the tetraploid cv. Otava, the number of identified ORFs was 153,000 [14] and the BUSCO completeness score was 97.3%. Six tetraploid genomes of European potato cultivars yielded the number of gene models ranged from 103,000 to 180,000 [15], which is comparable to the number of identified genes in the cv. Otava genome. On average, the number of identified protein-coding genes for the cv. Otava tetraploid genome assembly turns out to be about twice as high as our assemblies. This comparison demonstrates that protein-coding genes are underrepresented in our assemblies. This implies that the information about the functional characteristics and the abundance of proteins of different functional classes could be obtained only roughly from our data.

### 3.2. Protein Coding Genes Identification and Analysis

Short reads do not allow accurate reconstruction of the genomic sequences, which makes it difficult to estimate the conserved and variable parts of the pan-genome. For our assemblies, core and softcore genes account for approximately 60% of individual genomes, shell ~38%, and cloud ~2%. Assembling and analyzing six genomes of commercial potato cultivars with haplotype resolution resulted in twice the estimate of the proportion of core genes, ~80% [15], shell genes comprise 19%, and cloud 1–2%. Thus, our results underestimate the fraction of core genes in comparison with full tetraploid assemblies [6,15]. The difference in the two estimates is most likely due to the fact that our analysis did not allow separating similar genes belonging to different homologous chromosomes/haplotypes of potatoes.

Analysis of the pan-genome for our cultivars demonstrated its size did not reach a plateau. On the one hand, this can be explained by the small number of genomes studied: for diploid potato species, a plateau is reached when the number of genomes is close to 40 [6]. On the other hand, it can be partially explained by the high complexity of tetraploid potato genomes by gene content, expression, and function [15].

Despite the indicated inaccuracies, the general picture of the distribution of gene functions in core/shell parts of the pan-genome is similar to the results of plant pan-genome analysis from other works [6,15,28,29,30]: the variable part is more associated with genes of immune response and response to environmental stress conditions, the conserved part is primarily related to basic genome functions. In our work, the conservative part of the pan-genome of cultivars grown in Russia is enriched in genes involved in plant growth and development (PPR repeats, RNA recognition motifs, etc.), while the more variable part of the pan-genome is enriched in genes associated with plant immunity.

### 3.3. Diversity of the NBS-LRR Genes

*R*-genes mediate the plant response to various pathogens and pests. The diversity of immune response genes in potatoes is the basis for the development of new improved cultivars resistant to biotic stresses [31,32,33]. Reconstruction of potato genomes and transcriptomes reveals sequences encoding *R*-genes for further study [34,35]. The average number of complete NBS-LRR proteins in Russian potato cultivar genomes is ~230. Note that in the DM1-3 genome, 257 NBS-LRR genes were identified [34,36]. Estimates of the number of NBS domains alone for several dozen potato cultivars based on nucleotide marker alignment are about twice that number, 575–590 [35]. Due to the incompleteness of the genomic sequences of Russian potato cultivars, our estimates on the number of proteins belonging to different classes of NBS-LRR proteins determined for the reference DM1-3 genome are imprecise (Table 4). Some proteins belonging to specific classes are missed. For example, no proteins were detected at all for some classes in several Russian potato cultivars. However, we detected sequences dissimilar with annotated NBS-LRR proteins from DM1-3 (not falling into corresponding orthogroup clusters). Clearly, they represent variable parts of the NBS-LRR proteins in Russian potato cultivars and their fraction is quite large (~60%). This may indicate that potato accessions contain a diverse pool of *R*-genes, a significant proportion of which are unique to specific genomes, which is consistent with our results obtained earlier [34].

### 3.4. Study of Genetic Differentiation and Diversity of Cultivated Potato Genomes

The analysis of the genetic similarity between Russian and South American accessions showed that three Andean highlands “bitter” species (JUZ, CUR, AJH) separated from other accessions used in our study. This is consistent with the results of [3] demonstrated separation of these species into a separate cluster in the tree based on SSRs for landraces of all cultivated species and closely related wild species progenitors. In the tree obtained by [3], species *S. juzepczuki*, *S. curtilobum,* and *S. ajanhuiri* lie at the tree root, in relation to other representatives of cultivated potatoes. Specimens from these three species form separate clusters in the tree for potato germplasm collections at the Vavilov Institute of Plant Genetic Resources [37]. Thus, in agreement with [3,37], our results support the distinctive nature of hybrid species *S. curtilobum*, *S. juzepczukii,* and *S. ajanhuiri* within the group of cultivated potatoes [22].

The tree (Figure 3) also clearly distinguishes two sister clusters: the representatives of the *S. tuberosum* Andigenum group and the analyzed subset of improved potato cultivars grown in Russia. One possible reason for the separation of Russian potato cultivars and native South American accessions may be the involvement of Mexican wild species *S. demissum* and/or *S. stoloniferum* in the lineages of almost all Russian cultivars analyzed in this work (see data on molecular markers and references to the literature in the Supplementary File S4). This grouping is in agreement with the results of the phylogenetic tree of potatoes and its relatives (wild species, landraces, and improved cultivars) reconstructed using SNPs [38]. Note that in a diverse subset of Russian cultivars, we did not observe genetic differentiation (Figure 3) corresponding to their pedigrees (see references in the Supplementary File S4). Notably, in Figure 3 two clusters can be distinguished which include cultivars with contrasting characters in terms of their adaptability. One is the cluster of cvs. Zhukovsky, Nevsky, and Udacha (they hav been released in 10, 12, and 9 regions of Russian Federation, respectively, demonstrating a wide range of adaptability). The second cluster includes cvs. Symphonia, Severnoe Siyanie, and Fritella (released in 1, 1, and 2 regions, respectively, demonstrating narrow range of adaptability). This may reflect the genetic similarity between some cultivars with similar ecological diversification patterns.

### 3.5. CNVs Characteristics of Potato Cultivars

In our work, within subset of South American specimens there is only one of them representing a Chilean cultivar (TBR) [17]. This allowed us to evaluate the diversity and characteristics of CNV in Russian potato cultivars and to compare them with those of South American landraces genomes. The data were in agreement with previous results of potato CNV analysis [11,17,18,19,20,39]. The number of deletions is greater than the number of duplications in most of the accessions we studied (except for South American accessions of “bitter” cultivated species CUR and JUZ), including all cultivars from Russia. At the same time, the number of genes that underwent deletion is generally less than the number of duplicated genes (except South American diploid Andean landraces of STN, PHU, GON2, and GON1). This was also observed for South American landraces in [17], in the analysis of potato somatic hybrid, parents and progeny [19], diploid potato clones [20], and one of three potato cultivars of the Ural selection [39].

For the Russian potato cvs. Grand and Symphonia, the number of deleted genes is less than the number of duplicated genes slightly. In general, cultivars from Russia are more homogeneous in the characteristics of deletions and duplications, which seems reasonable, due to their genetic similarity (Figure 3). A similar pattern of relatively small variations in CNV was observed in the analysis of commercial tetraploid potato cultivars [18]. We also showed that CNV enrichment was observed in SAUR gene loci in both Russian and South American accessions, which is also consistent with earlier results [11,17].

The grouping of potato genotypes derived from the analysis of the two principal components derived from CNV (Figure 5 and Figure 6) is also consistent with the results of [17]: South American landraces of hybrid highland cultivated species AJH, CUR, JUZ are distant from other accessions that belong to *S. tuberosum* Andigenum group. Diploid Andean landrace accessions (diploids of *S. tuberosum* Andigenum group) GON1, GON2, STN, and PHU form a dense cluster, and tetraploid Andean landraces (ADG1 and ADG2) (tetraploids of *S. tuberosum* Andigenum group) are located next to each other. On the large scale, the PCA results are consistent with the genetic differentiation of these accessions (Figure 3): AJH, JUZ, and CUR accessions of ‘bitter’ potatoes group together; accessions of Andigenum Group (GON1, GON2, PHU, STN, CHA, ADG1, ADG2) fall into the second large cluster of South American accessions; Russian cultivars form the third large, dispersed group of accessions. It should be noted that these trees were obtained by different approaches (protein sequence similarity vs. CNV similarity) and differ by details (especially in relative positions of different accessions on the tree within groups of Russian and South American accessions). For example, in the PCA diagram, the TBR genotype is located close to the Russian potato cultivars, while they are distant on the genetic similarity tree. However, the clustering of this TBR accession (sample CIP 705053) with commercial potato cultivars having hybrid origin might be expected, because this TBR accession possesses the W-type plastome [40] which is characteristic for wild potato species and very seldom in potato landraces [41]. Whereas another cytoplasm type T (with 241 bp specific plastid deletion) is typical for Chilean landrace populations [41,42,43]. So, we can suppose that TBR accession (sample CIP 705053) is not a native Chilean landrace, it can be represented by an interspecific hybrid genotype. Interestingly, genetic analysis of the diversity and relatedness in an Andean potato collection from Argentina demonstrated, that some accessions classified as Andean landraces, consistently clustered with commercial cultivars supporting the hypothesis that they were, in fact, reintroductions of European-bred potato cultivars [44].

### 3.6. Patterns of CNV Occurrence in Russian and South American Cultivars

We distinguished the genes that are significantly different in CNV occurrence in genomes of Russian and South American accessions and investigated their function. In addition, we identified genes that have a predominance of CNV of the same type (duplications/deletions) in either Russian cultivars or South American landraces. This analysis allows us to evaluate the contribution of CNVs to the rate of diversification of improved cultivars grown in Russia and South American native cultivars (landraces) during their breeding process [21].

Functional analysis of the genes having different CNV frequencies in Russian and South American accessions showed several functional clusters of terms. Interestingly, some of them were also identified in the analysis of CNV patterns associated with duplications in Russian cultivars (RUDup). According to the similarity of the annotation terms, these clusters can be divided into three groups. First of all, these are plant immune response genes containing NB-ARC, LRR, and TIR domains (clusters 1,5,10). This is not surprising since immune response receptors in potatoes show a high level of diversity [19,34,35,45], which is also consistent with the results of the NBS-LRR sequence analysis (Table 4). A specific set of combinations of genes of resistance to particular pathogens could have been formed during the selection of these cultivars. Thus, this group of genes reflects the great diversity of immune response genes for South American and Russian potato accessions.

The second group represents genes involved in transport processes. These are vesicle-mediated transport/vacuolar sorting as well as voltage-gated anion channel activity, porin domain (clusters 4, 7). Vacuoles are known to serve as accumulators of secondary plant metabolites such as alkaloids, phenolic compounds, xenobiotics, etc. [46,47]. Porins are involved in the exchange of ions and small molecules across the mitochondrial outer membrane and engaged in complex interactions driving many facets of cell function [48]. Probably, the differences in CNV associated with these functions reflect the differences in the accumulation and transport of various metabolites in South American and Russian cultivars subsets.

The third group of genes (clusters 2, 3, 6, 8, 9) can be described by the general response to abiotic stresses and affecting other important processes in plants. For example, aspartic-type endopeptidases (cluster 6) have been associated with plant defense response mechanisms, hybrid sterility, reproductive development, abiotic and biotic stresses, chloroplast homeostasis, and lateral root development [48]. It has been shown that segmental and tandem duplications are characteristic of genes in this group in the potato genome, and the expression of approximately 21% of these genes changes under salt, osmotic, or temperature treatments [49]. Genes from the 3^rd^ functional cluster (replication factor A) are also shown to change expression associated with endoplasmic reticulum stress and potential involvement in genotoxic stress responses [50]. Genes from cluster 9 are involved in RNA methylation processes and related to the development and abiotic stress processes in plants [51]. Genes from cluster 8 (FBD domain) are also involved in plant development and stress response, ubiquitinylation processes [52]. The cluster 2 gene (Poly(ADP-ribose) glycohydrolase, gene ID PGSC0003DMG400029361), whose homolog in *Arabidopsis* (PARG) regulates immune gene expression and defense responses [53], can also be assigned to this group. In plants, unlike animals, these genes are present in the genome in multiple copies and are involved in a wide range of important biological processes [54]. These genes are also involved in the response to a number of abiotic stresses [54,55,56,57]. In general, the variability of the third group of genes is consistent with the processes of diversification of cultivated potato accessions and their adaptation to new growing conditions.

### 3.7. CNV Differences in Genes Related to Tuberization/Photoperiod

The presence of CNVs in genes associated with photoperiod in crops is an important factor in their diversification during adaptation to new climatic zones [21]. CNVs of a number of genes have been shown to affect flowering in wheat [58], and barley [59], flowering and heading time in winter wheat [60,61] photoperiod sensitivity in bread wheat [62], heading time in durum wheat [63].

Our analysis showed that genomes of Russian cultivars adapted to the long-day high northern latitudes and short-day adapted Andean landraces differ in their CNV occurrence for 4 of 48 known genes related to tuber formation and response to photoperiod changes (Table 5). One of them is phytochrome A, which is involved in circadian clock control in potatoes [26] and potentially involved in resistance to adverse factors [64], three were identified as differentially expressed in two tetraploid cultivars with short and long tuberization times [25].

In addition, we detected the poly(ADP-ribose) glycohydrolase gene (PARG, gene ID PGSC0003DMG400029361) (Table 5) located on chromosome 12. We identified significant duplications of this gene for all 15 Russian tetraploid cultivars, and only for 3 South American polyploid landraces (CHA, TBR, ADG2). Experiments show that poly(ADP-ribose) glycohydrolases in *Arabidopsis thaliana* play an important role in the regulation of circadian oscillator [27]: an inhibitor of PARG shortened the period length of wild-type plants. Interestingly, the function of this gene in potatoes in relation to the control of circadian rhythms has not been previously reported.

Our results reflect the importance of CNV in the adaptation of European and, in particular, Russian potato cultivars to the conditions of a longer photoperiod characteristic of higher latitudes in Europe and Russia [4,65]. However, we may suppose that the CNVs are not common for the most tuberization and photoperiod control genes (Supplementary File S3), and their variability seems to be described mostly by the accumulation of nucleotide replacements or short insertions/deletions in the genes themselves [66,67] or their upstream regions [15].

Thus, the comparative analysis of CNV occurrence in the genomes South American and Russian potato cultivars shows that structural variations are closely related to the processes of diversification in potato cultivars. First of all, these are well-known processes for cultivated plants: immune response, stress response, and processes of transport of metabolites and ions in cells. In addition, there are a number of specific processes, important for potato adaptation, such as the control of photoperiod or/and tuberization.

## 4. Materials and Methods

### 4.1. Plant Material

We analyzed the genomes of 15 potato cultivars grown in Russia. They include a subset of 14 improved (contemporary) potato cultivars with diverse breeding backgrounds developed in Russia (Supplementary File S4) [37,68,69]. The plants of 10 cultivars were obtained from the in vitro collection maintained at the N.I. Vavilov All-Russian Institute of Plant Genetic Resources (VIR), Saint Petersburg, Russia, and plants of the rest 4 cultivars were obtained from the in vitro collection GeneAgro of the Institute of Cytology and Genetics SB RAS, Novosibirsk, Russia. Names, details of pedigree (parental genotypes), information on the year of release, and place of origin (Breeding Centre or Company) are listed in Supplementary File S4. This file also provides links to external resources with more information on genotyping data (as DOI for the corresponding articles). These 14 cultivars previously were genotyped using nSSR fingerprinting; in addition, they were screened with 15 DNA markers associated with 10 *R*-genes conferring resistance to diseases and pests and their cytoplasm types were established using the commonly used set of organelle DNA-specific primers (Supplementary File S4).

These cultivars represent breeding programs conducted in different breeding centers located in the central part of Russian Federation, Moscow region (Russian Potato Research Centre; LLC Agrocenter “Korenevo”) and in the north-west part of Russian Federation, Saint Petersburg region (Leningrad Research Agriculture Institute — Branch of Russian Potato Research Centre; LLC Selection firm “LIGA”). Fourteen Russian cultivars represent different maturity types and ranges of use (table, starch, and processing potato cultivars). They were selected to represent a wide range of genetic diversity and origin that had been identified based on previous results of molecular studies and pedigree information (Supplementary File S4).

These cultivars can be divided into three groups according to their cytoplasm types which are corresponded to their maternal parentage. Group 1: four cultivars (Grand, Gusar, Meteor, Sudarinya) have W/gamma type cytoplasm that is typical for interspecific hybrids with wild Mexican species *S. stoloniferum* × *S. tuberosum*. Group 2: seven cultivars (Fritella, Krasa Meshchery, Krepysh, Nevsky, Nikulinsky, Udacha, Zhukovsky ranny) have the D-type cytoplasm as known from the results of molecular analysis; the D-type cytoplasm is typical for wild Mexican species *S. demissum*. Group 3: three cultivars (Golubizna, Krasavchik, Severnoe siyanie)— typical for Chilean *S. tuberosum* cytoplasm type T-W wild species were involved in their pedigrees as paternal parents.

In addition to 14 Russian cultivars, 1 foreign Dutch cv. Symphonia was also involved in our study. This cultivar is characterized by high resistance to wart disease, potato cyst nematode, and moderate resistance to scab, but is susceptible to late blight. It is actively used in breeding and genetic research in Russia [70,71,72,73,74]. We use term “Russian cultivars” for all these 15 cultivars, including Symphonia for simplicity in this paper.

These cultivars demonstrate very broad adaptability (ecological plasticity) under different climatic and ecological conditions (Supplementary File S4). For example, cvs. Nevsky, Zhukovsky, and Udacha have been approved for 12, 10, and 9 regions of the Russian Federation, respectively, relating to the contrasting light zones (The State Register of Selection Achievements Approved for Use, in Russian, https://reestr.gossortrf.ru/, accessed on 2 March 2023). On the other hand, there are cultivars that have been released only in one particular region of the Russian Federation (for example, cvs. Krasavchik and Severnoe Siyanie); the Dutch cv. Symphonia is among them (approved only for one region of the Russian Federation (Supplementary File S4). Since the number of narrow adaptive cultivars in this study was small, we included Symphony in the analysis as well.

Most Russian cultivars were registered in the VIR Genebank both as living accessions and as nomenclature standards in the form of an herbarium voucher according to the International Code of Nomenclature for Cultivated Plants (ICNCP) [75]. Supplementary File S4 includes information about code of the herbarium vouchers (nomenclatural standards) which are maintained in the WIR Herbarium of cultivated plants, and their wild relatives located in Saint Petersburg. The plant material was provided to the WIR Herbarium by the authors of the cultivars.

### 4.2. DNA Samples Preparation and Sequencing

Green leave samples of 0.1–0.15 g were frozen in liquid nitrogen. The frozen leaves were ground in a ceramic mortar with the addition of liquid nitrogen. Using a cold spatula, the ground mass was transferred to a 1.5 mL test tube. DNA extraction was performed using a DNeasy Plant Mini Kit, QIAGEN (Germany), according to the instructions. An amount of 400 μL of AP1 solution and 4 μL of RNase A (100 mg/mL) were added to the ground mass. The mixture was then incubated in a water bath at 65 °C for 10 min. After that, 130 µL of P3 solution was added, stirred, and incubated on ice for 5 min. After standing on ice, it was centrifuged for 5 min at 14,000 rpm. The lysate was transferred to a QIAshedder Mini spin column, then centrifuged again for 2 min at 14,000 rpm. The purified lysate was carefully transferred to a 1.5 mL tube, where it was mixed with 1.5× volume of AW1 solution and pipetted. The resulting solution was filtered on DNeasy Mini spin columns by centrifugation for 1 min at 8000 rpm. The columns were then transferred to a new tube, and double purification with AW2 solution was performed. DNA elution was performed with AE solution with a total volume of 150 µL in two steps.

Two mkg DNA from potato leaves was fragmented using a Covaris M220 sonicator with parameters optimized for a maximum fragment size of approximately 400 bp for library preparation. Barcoded genome libraries were prepared using 100 ng of fragmented DNA, with Roche KAPA Hyper Prep Kit, and KAPA UDI adapters (ROCHE, Basel, Switzerland), according to the manufacturer’s protocol for dual size selection. Nine PCR cycles were used for amplification, followed by AMPure XP (Agencourt, Brea, CA, USA) purification. Final libraries quantification was performed with a Bioanalyzer 2100 and a DNA High Sensitivity Kit (Agilent, Santa Clara, CA, USA). After normalization, barcoded libraries were pooled and sequenced on a NextSeq 550 or Novaseq 6000 platform (Illimina, San Diego, CA, USA) with 2 × 150 bp paired-end reads. The *.bcl files were converted to fastq format and demultiplexed using the bcl2fastq software (https://support.illumina.com/sequencing/sequencing_software/bcl2fastq-conversion-software.html, accessed at 21 January 2022) according to the developer’s instructions.

### 4.3. Reference Genome and Sequences of South American Potato Landraces

We used the DM1-3 v4.04 assembly [8], which was downloaded from the SpudDB database—Potato Genomic Resources (http://spuddb.uga.edu/, accessed on 12 February 2022), as the reference genome of potato *S. tuberosum*. AGAT v. 0.9.2 [76] was used to obtain the amino acid sequences of the DM1-3 reference genome based on DM1-3 genome annotation v4.03. The amino acid sequences of tomato *S. lycopersicum* SL3.0 [77] downloaded from the Ensembl Plants database (http://plants.ensembl.org, accessed on 12 February 2022) were used as an outgroup for the analysis of genetic diversity in potato accessions studied.

We obtained a library of potato transposable elements using the EDTA package [78] and the DM1-3 reference genomic sequence. This library was used to further identify TEs in the contigs we assembled.

The sequences of South American *S. tuberosum* potato genomes [17] were downloaded from NCBI by BioProject identifier PRJNA556263. Raw data from SRA: SRR10244436 (BUK), SRR10244437 (AJH), SRR10244438 (STN), SRR10244439 (PHU), SRR10244440 (GON2), SRR10244441 (GON1), SRR10248510 (CUR), SRR10248511 (CHA), SRR10248512 (JUZ), SRR10248513 (TBR), SRR10248514 (ADG2), SRR10248515 (ADG1). Genome assemblies from NCBI genomic database: ASM984962v1 (CHA), ASM984964v1 (CUR), ASM984968v1 (JUZ), ASM984970v1 (ADG1), ASM984972v1 (ADG2), ASM984974v1 (TBR), ASM984975v1 (PHU), ASM984978v1 (STN), ASM984980v1 (AJH), ASM984981v1 (BUK), ASM984986v2 (GON1), ASM984990v1 (GON2). Description of the South American accessions is shown in Table S4 (Supplementary File S1).

### 4.4. Genome Assembly and Quality Estimation

Genomic sequences of potato varieties cultivated in Russia were processed using the following bioinformatics pipeline:

1. Genome assembly up to contig level using MaSuRCA v3.4.2 [79];
2. Assembly quality estimation using QUAST v5.2.0 [80];
3. TEs masking using obtained TE libraries and RepeatMasker (http://www.repeatmasker.org/, accessed 2 February 2020) [81];
4. ORF identification using AUGUSTUS v3.4.0 [82];
5. Filtering contigs with length below 1000 bps not containing ORFs;
6. Evaluation of the genome completeness performed using BUSCO v5.3.0 [83] with Solanales DB10 (5 August 2020).

The open reading frames for South American potato genomic sequences identified these genomes using AUGUSTUS v3.4.0 [82], see step 4 of the pipeline.

### 4.5. Orthologous Gene Groups Identification and Genetic Diversity Analysis

Identification and analysis of orthologous groups were performed for protein-coding sequences of 15 cultivars grown in Russia, 12 South American potato accessions, the potato reference genome DM1-3 and the outgroup *S. lycopersicum* tomato genome.

OrthoFinder v2.5.2 [84] was used to identify orthologous groups. This program was also used to build a tree reflecting genetic diversity between potato accessions. OrthoFinder was run with the -m MSA parameter, which allowed us to reconstruct the species tree using an algorithm based on the reconstruction of phylogenetic trees of individual orthogroups and their combinations. This method takes into account possible duplications and loss of genes within individual orthogroups, which is important in our case of genomic sequences reconstructed from short reads. The phylogenetic tree was visualized using iTOL v.6 [85].

### 4.6. Orthologous Gene Groups Identification and Phylogenetic Reconstruction

The orthologs for genomes of 15 Russian cultivars were classified into 4 classes: core (the orthogroup includes sequences from all 15 genomes), softcore (the orthogroup includes sequences from 14 genomes), shell (the orthogroup includes sequences from 2–13 genomes), and cloud (the orthogroup includes sequences from single genome). Pan-genome size modeling based on orthogroup data was performed via PanGP v1.0.1 [86] with a random algorithm and sample size of 500.

Functional annotation of proteins from each genome for Russian and South American accessions was performed using InterProScan v5.51.85 [87]. The frequency of occurrence of a particular protein function term was calculated based on annotation of Pfam domains [88] and InterPro database identifiers. Next, the frequency of occurrence of the protein function term in the variable and conserved parts of the potato pan-genome was estimated.

### 4.7. Identification and Analysis of NBS-LRR Genes

NBS-LRR domains of proteins associated with plant immunity were searched in the amino acid sequences of the genomes of Russian potato cultivars. The NLR-Parser program was used for this purpose [89]. Only those proteins in which the complete (“complete”) and true (“true”) domain structure of NLR proteins was reported by NLR-Parser were taken for further consideration. To classify these sequences into different classes (TNL, CNL-R and CNL1-8 groups), we used the partitioning of protein sequences into orthogroups (see Section 4.5 above). The orthogroup sequences in which the corresponding classes of NBS-LRR proteins of the potato reference genome DM1-3 [36] were represented were assigned to these classes. The partitioning of the reference genome proteins into the corresponding classes was taken from ref. [36] (listed also in the Supplementary file “Identical_NB_ARC.xlsx” from ref. [34]).

### 4.8. CNV Identification and Analysis

Illumina short reads were used to evaluate and analyze Copy Number Variation in Russian and South American potato genomes. Quality filtering of reads and trimming of adapters was performed using Trimmomatic v0.39 [90] with the following parameters: TruSeq3-PE.fa:2:30:10 SLIDINGWINDOW:5:20 LEADING:20 TRAILING:20 MINLEN:50. Trimmed reads were aligned to the DM1-3 v4.04 reference gene using BWA v0.7.17 [91]. Reads were labeled for duplicates via Picard v2.26.1 (https://broadinstitute.github.io/picard/, accessed 2 February 2020) and sorted and indexed using SAMTOOLS v1.12 [92]. Only properly paired reads were used for further analysis.

Alignment results were used to detect CNV using CNVpytor v0.4.1 [93]. CNVs were detected on all chromosomes of the DM1-3 v4.04 reference genome as well as on pseudomolecules (chr00 and ChrUn). CNVs calls were filtered as follows: length greater than 1 kb, P-value (first e-value) < 0.01, q0 < 50%, and pN < 50%. The R package intansv v1.12.0 [94] was used to find correspondence between the identified CNVs and the genes in the potato genome. For this purpose, the CNVpytor output files were converted to the format required for input by removing the last two columns (pN and dG).

Visualization of the position of CNVs in the DM1-3 genome for Russian and South American cultivars was obtained using the Circos [95].

The list of CNVs was formatted as a table with rows corresponding to the potato genotype and columns corresponding to the genes for which significant CNVs were identified for further statistical processing. The table element was +1 if the CNV corresponded to a duplication of a region, -1 if the CNV was a deletion, and 0 if no significant CNVs for the accession were found in that region.

To evaluate the similarity of Russian and South American potato accessions by their CNV characteristics, we performed principal component analysis (PCA) using column of the above table using as variables by the Scikit-learn v1.1.2 package [96]. A tree for potato genomes based on their CNV similarity was built using the PARS algorithm of PHYLIP [97].

In order to identify CNVs with significant differences in occurrences within the groups of Russian and South American potato genomes, we used the analysis of 2 × 3 contingency tables for the table of CNV +1/0/-1 types for genes described above. Genotypes were classified into 2 classes: Russian and South American. We considered eight South American accessions (CHA, ADG1, ADG2, TBR, PHU, STN, GON1, GON2) forming a sister cluster in relation to genomes of Russian cultivars analyzed here. CNVs were classified into three types: −1, +1, 0 (see above). The significance of associations between potato variety type and CNVs was assessed using Fisher’s exact test implemented in the Python rpy2 library (https://rpy2.github.io/, accessed 02.07.2021). The association between CNV and genome types was considered significant at a *p*-value < 0.01. Additionally, we classified genes with CNV according to specific patterns of representation in potato variety groups. RUDup: genes with significant duplications detected in 50% or more of Russian cultivars, while they are absent in South American accessions. RUDel: genes that have significant deletions in 50% or more of Russian cultivars but are absent in South American accessions. SADup: genes that have significant duplications in 50% or more of South American accessions, but do not have them in Russian cultivars. SADel: genes found to have significant deletions in 50% or more of South American accessions, but not in Russian cultivars.

Functional analysis of gene groups with specific CNV patterns was performed using the DAVID web service [98].

Adaptation to the long day period is an important diversification factor for potato varieties. Therefore, we compiled a list of genes related to tuberization and photoperiodicity processes in potatoes and searched these genes among those having specific CNV patterns in populations of genomes of Russian cultivars and South American accessions analyzed in the present study. For this purpose, the initial list of tuberization and photoperiodicity-related genes was taken from a review [24] based on references given there [67,99,100,101,102,103,104,105] and supplemented with genes from refs. [25,26]. A total of 48 potato genes were included in the list (Supplementary File S3).

## 5. Conclusions

In the present work, we performed short-read sequencing, assembly, and structural analysis of genomes of 15 cultivars grown in Russia (14 cultivars of Russian origin and 1 Dutch cultivar). The main characteristics of our assemblies are consistent with those obtained from the assemblies for short reads of various potato genomes (~60% of the genes belong to the conserved part of the pan-genome, 38% to the shell, and 2% to the cloud part). ORFs from the pan-genome core are related to basal gene function, the variable part is associated with immune response, and genes are responsible for environmental adaptation. The set of the NBS-LRR genes in the accessions is highly variable: on average we identified 227 complete NBS-LRR sequences per genome; the fraction of classified genes into CNL/TNL classes is about 40%.

South American potato genomes included in our comparative analysis demonstrated separation from the Russian potato genomes on the genetic differentiation tree: highland Andean (*S. juzepczukii*, *S. curtilobum*, and *S. ahanhuiri*), other South American and Russian accessions. We performed CNV analysis, the results of which on the distribution of their main characteristics in Russian and South American accessions are in agreement with previously published data: the number of deletions exceeds the number of duplications, with a higher number of genes with duplications than with deletions. Russian cultivars demonstrated homogeneity in CMV characteristics in comparison with South American potato landraces.

We identified genes with CNV with different occurrences in South American and Russian potato accessions. This allowed us to evaluate the functions of the genes associated with the diversification of Russian and South American potato cultivars. The functional analysis of these genes showed that a significant part of them is related to the immune response or response to abiotic stress. At the same time, a detailed analysis of genes related to tuberization, and photoperiod control revealed significant differences in CNV occurrence in four of the known genes and identified an additional gene homologous to the PARG gene of *Arabidopsis*, which may be involved in circadian rhythm control processes related to the acclimation processes of Russian potato cultivars.

## Figures and Tables

**Table 1:** Genome assembly statistics of potato varieties cultivars grown in Russia after filtering of short contigs (<1000 bp) without ORFs.

Cultivar	Abbreviation	Contig Number	Assembly Size, bp	GC, %	Largest Contig Length, bp	N50	L50	Total Repeats, %
Fritella	Fri	163,562	1,222,793,321	35.10	212,769	15,379	19,162	61.15
Golubizna	Golu	257,855	960,248,392	35.83	210,050	9555	17,354	60.82
Grand	Grand	155,461	1,170,073,532	35.21	246,541	15,505	18,186	60.86
Gusar	Gus	269,494	938,553,975	36.22	178,581	8095	20,020	61.70
Krasa Meshchery	KrMe	217,519	1,297,174,757	35.54	162,815	12,476	23,869	62.65
Krasavchik	Kras	202,187	1,196,647,232	35.61	192,449	12,886	20,742	61.68
Krepysh	Krep	252,102	994,694,667	35.58	280,787	9148	19,978	61.84
Meteor	Met	242,362	877,113,923	35.59	164,801	9975	16,041	60.46
Nevsky	Nev	209,314	843,177,798	35.63	176,082	11,816	13,716	60.45
Nikulinsky	Nik	193,033	1,149,833,873	34.83	204,478	11,649	22,013	60.75
Severnoe Siyanie	SevS	207,781	1,260,128,866	35.64	222,665	13,423	21,160	62.38
Sudarinya	Sud	259,781	894,173,936	36.03	204,234	9426	16,513	61.34
Symphonia	Symph	176,350	1,119,406,986	34.92	216,399	12,272	21,685	61.08
Udacha	Uda	204,824	652,821,260	36.09	236,853	8151	14,114	59.72
Zhukovsky	Zhu	214,788	899,426,469	35.47	202,351	12,049	14,400	60.63

**Table 2:** Orthogroup statistics for ORFs from the genomes of Russian potato cultivars, South American accessions, potato DM1-3, and *S. lycopersicum*.

Accession	ORF Number	Number of ORFs in Orthogroups	Fraction of ORFs in Orthogroups, %	Number of Unassigned ORFs	Fraction of Unassigned ORFs, %
Subset of Russian cultivars:					
Fritella	73,209	72,212	98.6	997	1.4
Golubizna	72,457	71,319	98.4	1138	1.6
Grand	70,304	68,989	98.1	1315	1.9
Gusar	71,079	69,890	98.3	1189	1.7
Krasa Meshchery	77,417	76,041	98.2	1376	1.8
Krasavchik	73,815	72,545	98.3	1270	1.7
Krepysh	68,362	67,432	98.6	930	1.4
Meteor	68,165	67,080	98.4	1085	1.6
Nevsky	62,916	61,934	98.4	982	1.6
Nikulinsky	73,667	72,727	98.7	940	1.3
Severnoe Siyanie	75,701	74,316	98.2	1385	1.8
Sudarinya	68,864	67,563	98.1	1301	1.9
Symphonia	69,783	68,936	98.8	847	1.2
Udacha	60,411	59,416	98.4	995	1.6
Zhukovsky	64,386	63,352	98.4	1034	1.6
Subset of South American accessions ^1^:					
AJH	75,555	74,644	98.8	911	1.2
BUK	101,267	98,907	97.7	2360	2.3
CHA	76,301	75,521	99.0	780	1.0
PHU	79,451	78,715	99.1	736	0.9
GON1	55,935	55,397	99.0	538	1.0
GON2	83,068	80,518	96.9	2550	3.1
STN	88,448	87,334	98.7	1114	1.3
TBR	119,470	114,616	95.9	4854	4.1
ADG1	47,969	47,822	99.7	147	0.3
ADG2	95,322	93,627	98.2	1695	1.8
CUR	119,710	116,691	97.5	3019	2.5
JUZ	92,022	90,686	98.5	1336	1.5
*S.tuberosum* DM1-3 v. 403	39,028	37,828	96.9	1200	3.1
*S. lycopersicum*	34,429	31,159	90.5	3270	9.5

^1^ Subset of 12 South American accessions includes representatives of *S. tuberosum* Andigenum group (STN, PHU, GON1, GON2, CHA, ADG1, ADG2), *S. tuberosum* Chilotanum group (TBR), *S. ajanhuiri* (AJH), *S. curtilobum* (CUR), *S. juzepczukii* (JUZ), *S. bukasovii* (BUK) [17], see also Materials and methods Section 4.3.

**Table 3:** The number and fraction of protein-coding genes in genomes of Russian cultivars annotated by InterproScan program.

Cultivar	Number of Functionally Annotated ORFs	Fraction of Functionally Annotated ORFs, %
Fritella	42,187	58.42
Golubizna	38,428	53.88
Grand	40,731	59.04
Gusar	37,170	53.18
Krasa Meshchery	40,978	53.89
Krasavchik	42,144	58.09
Krepysh	36,791	54.56
Meteor	36,323	54.16
Nevsky	34,391	55.53
Nikulinsky	40,840	56.16
Severnoe siyanie	41,933	56.42
Sudarinya	36,386	53.85
Symphonia	39,012	56.59
Udacha	32,247	54.27
Zhukovsky	35,344	55.79

**Table 4:** Number of full-length NBS-LRR genes of different classes identified in genomes of Russian potato cultivars and reference genome DM1-3.

Cultivar	CNL Type									TNL	n/a	Total
	1	2	3	4	5	6	7	8	R			
Fritella	27	4	7	0	7	8	21	3	20	15	190	302
Golubizna	15	4	0	0	3	7	14	2	12	12	118	187
Grand	29	6	1	1	5	11	23	1	13	17	191	298
Gusar	13	4	2	1	4	3	18	0	9	11	117	182
Krasa Meshchery	24	5	3	0	5	10	12	5	11	19	148	242
Krasavchik	15	9	7	2	11	9	17	3	15	12	181	281
Krepysh	21	4	5	2	3	5	13	3	12	14	147	229
Meteor	17	3	1	0	6	10	19	1	10	13	112	192
Nevsky	12	4	0	1	7	11	14	2	11	12	111	185
Nikulinsky	15	6	6	0	3	8	17	5	17	11	163	251
Severnoe Siyanie	15	8	2	2	11	10	25	4	17	20	192	306
Sudarinya	19	4	1	0	3	5	14	2	12	11	110	181
Symphonia	25	5	3	1	5	11	17	2	17	14	152	252
Udacha	11	3	1	1	4	6	12	0	10	4	83	135
Zhukovsky	9	5	2	0	3	5	15	1	12	10	119	181
Total by class	267	74	41	11	80	119	251	34	198	195	2134	3404
*S.tuberosum* DM1-3	16	13	20	9	31	23	23	24	32	66	0	257

**Table 5:** The list of genes associated with tuberization and photoperiod control, for which CNV occurrence in genomes of Russian and South American accessions is significantly different. Gene IDs, number of significant CNV of deletion/duplication types in compared subsets, *p*-values (Fisher’s test), and gene description are given.

Gene ID	RU del/dup	SA del/dup	*p*-Value	Gene Description
PGSC0003DMG400000678	0/2	4/1	0.005	Metallocarboxy-peptidase inhibitor ^1^
PGSC0003DMG400012838	4/0	7/0	0.009	Non-specific lipid-transfer protein ^1^
PGSC0003DMG400023272	0/0	4/0	0.008	Elongation factor 1-alpha ^1^
PGSC0003DMG400015766	1/8	4/0	0.010	Phytochrome A ^2^
PGSC0003DMG400029361	0/15	0/3	0.002	Poly(ADP-ribose) glycohydrolase ^3^

^1^ See ref. [25], ^2^ See ref. [26], ^3^ This work.

## Data Availability

The assembled genomes and all raw sequencing data have been deposited under NCBI BioProject PRJNA933976.

## Supplementary Materials

The supporting information can be downloaded at: https://www.mdpi.com/article/10.3390/ijms24065713/s1. References [3,4,17,22,106,107] are cited in the supplementary materials.

## Abbreviations

CNV	Copy number variation
cv.	Cultivar
NBS-LRR	Nucleotide-binding site and leucine-rich repeats
ORF	Open reading frame
PC	Principal component
PCA	Principal component analysis
SAUR	Small auxin-up RNA
