# Supplementary material for: Analysis of Genome Structure and Its Variations in Potato Cultivars Grown in Russia
Source: Int J Mol Sci. 2023 Mar 16;24(6):5713. doi: 10.3390/ijms24065713 (PMC10059000; doi:10.3390/ijms24065713)
Supplement: Supplementary file 1 [file ijms-24-05713-s001.zip › Supplementary File S1.pdf]

Supplementary file 1 for Karetnikov et al. "Analysis genome structure and its variations for potato cultivars grown in Russia", International Journal of Molecular Sciences

Table S1. Basic library statistics for *S. tuberosum* cultivars from Russia.

Cultivar	Abbreviation	Number of reads	DM1-3 coverage, ×	Number of paired reads after trimming, %
Fritella	Fri	280,935,812	104	87
Golubizna	Golu	124,191,124	46	91
Grand	Grand	293,713,517	109	88
Gusar	Gus	129,467,175	48	91
Krasa Meshchery	KrMe	234,792,420	86	87
Krasavchik	Kras	292,082,433	108	83
Krepysh	Krep	137,019,167	51	92
Meteor	Met	110,142,606	41	91
Nevsky	Nev	78,862,788	29	95
Nikulinsky	Nik	162,266,560	60	100
Severnoe Siyanie	SevS	291,945,181	108	87
Sudarinya	Sud	90,320,534	33	92
Symphonia	Symph	183,625,275	68	91
Udacha	Uda	62,316,347	23	93
Zhukovsky	Zhu	92,549,685	34	94

Table S2. Statistics on identified CNVs in genome assemblies of Russian and South American potato accessions.

Accession	Number of deletions	Number of duplications	Total number of CNVs	Mean CNV length, bp	Largest size of deletion, bp	Largest size of duplication, bp	Largest size of CNV, bp	Number of genes with deletion	Number of genes with duplication	Number of genes with CNV
Fri	23175	10252	33427	7452	141200	106500	141200	5057	11172	16229
Golu	21015	13461	34476	9449	136900	128800	136900	4658	15481	20139
Grand	22782	7023	29805	6595	114100	67100	114100	4961	4800	9761
Gus	18647	12554	31201	9299	114100	125600	125600	4099	13562	17661
Kras	21738	12912	34650	8426	107500	162800	162800	4821	13938	18759
Krep	20730	10568	31298	8366	181300	130300	181300	4722	12275	16997
KrMe	22149	9663	31812	7569	137000	105800	137000	4710	6898	11608
Met	20573	10859	31432	9166	126900	158800	158800	4755	9761	14516
Nev	19487	10941	30428	8912	396200	116900	396200	4599	9678	14277
Nik	22910	9270	32180	8016	141000	102500	141000	5147	7115	12262
SevS	21062	11453	32515	8251	120400	148600	148600	4636	10184	14820
Sud	20596	13933	34529	9533	176200	148800	176200	4666	16273	20939
Symph	21631	8099	29730	7385	114100	90000	114100	5017	4615	9632
Uda	18280	13864	32144	10506	250100	160800	250100	4337	16804	21141
Zhu	20318	10882	31200	8895	220200	137900	220200	4845	10408	15253
ADG1	18182	10515	28697	9112	283800	135700	283800	4362	9494	13856
ADG2	20863	8286	29149	7737	683700	92100	683700	5199	6402	11601
AJH	20024	19364	39388	9488	396500	157900	396500	4767	20945	25712
BUK	24400	13775	38175	9760	815200	149000	815200	6731	14620	21351
CHA	21792	13899	35691	9116	582400	121700	582400	5643	11982	17625
CUR	15415	19640	35055	11601	589600	147700	589600	3568	24910	28478
GON1	19661	4031	23692	6806	305500	56000	305500	4974	2036	7010
GON2	19512	3426	22938	6130	176300	50500	176300	4520	1551	6071
JUZ	16272	20043	36315	12038	606200	175200	606200	3658	27292	30950
PHU	21348	5966	27314	7319	646500	70100	646500	5697	3485	9182
STN	23543	6488	30031	7404	452400	104500	452400	6100	3711	9811
TBR	19604	14314	33918	9689	646500	137000	646500	5244	15073	20317

Table S3. Tests for a difference in means and the equality of two variances for various CNV characteristics in Russian cultivars (RU) and South American accessions (SA). The table shows *p*-values for corresponding characteristics. Significant (<0.05) *p*-values shown in bold. Number of Russian cultivars is 15; number of South American cultivars is 12.

CNV characteristic	RU mean	RU variance	SA mean	SA variance	*t*-tests for a difference in means, *p*-values	*F*-test of the equality of two variances, *p*-values
Number of deletions	21006	2.13×10^{6}	20051	6.94×10^{6}	0.24	**0.04**
Number of duplications	11049	4.21×10^{6}	11646	3.71×10^{7}	0.72	**3.13×10^{-04}**
Maximum deletion size	1.65×10^{5}	5.81×10^{9}	5.15×10^{5}	3.65×10^{10}	**8.17×10^{-07}**	**1.95×10^{-03}**
Maximum duplication size	1.26×10^{5}	7.88×10^{8}	1.16×10^{5}	1.71×10^{9}	0.48	0.17
Number of genes with deletions	4735	73329	5038	8.85×10^{5}	0.24	**5.06×10^{-05}**
Number of genes with duplications	10864	1.53×10^{7}	11792	8.03×10^{7}	0.72	**4.77×10^{-03}**

Table S4. Information about 12 South American accessions from Kyriakidou et al. [3] (see Literature, p. 9 of this document)

Species,<br>the latest taxonomic treatment of Spooner et al. [4,5,6]	Species,<br>the most acceptable taxonomic treatment of Hawkes, 1990 [2]	Species,<br>taxonomic treatment of Dodds, 1962 [1]	Abbreviation according to Kyriakidou et al., 2020 [3]	Accessions used by Kyriakidou et al., 2020 [3]
Wild ancestor species:				
S. candolleanum<br>Berthault.	***S. bukasovii*** Juz.<br>2n = 2x	***S. bukasovii***<br>2n = 2x	BUK	CIP 761748
Andean cultivated highland bitter potato species:				
S. curtilobum Juz. & Bukasov<br>2n = 5x	***S. curtilobum*** Juz. & Bukasov<br>2n = 5x	***S.× curtilobum***<br>2n = 5x	CUR	CIP 702937
S. juzepczukii Bukasov<br>2n = 3x	***S. juzepczukii*** Bukasov<br>2n = 3x	***S.× Juzepczukii***<br>2n = 3x	JUZ	CIP 706050
S. ajanhuiri Juz. & Bukasov<br>2n = 2x	***S. ajanhuiri*** Juz. & Bukasov<br>2n = 2x	-	AJH	CIP 703810
Andean cultivated potato species:				
S. tuberosum		***S. tuberosum***		
S. tuberosum Andigenum group<br>2n = 2x	***S. stenotomum*** Juz. & Bukasov<br>2n = 2x	Group Stenotomum<br>2n = 2x	STN	CIP 705834
S. tuberosum Andigenum group<br>2n = 2x	S. *stenotomum* subsp. *goniocalyx* (Juz. & Bukasov) Hawkes<br>2n = 2x	Subgroup Goniocalyx<br>2n = 2x	GON1<br>GON2	CIP 702472<br>CIP 704393
S. tuberosum Andigenum group<br>2n = 2x	***S. phureja*** Juz. & Bukasov<br>2n = 2x	Group Phureja<br>2n = 2x	PHU	CIP 703654
S. tuberosum Andigenum group<br>2n = 3x	***S. chaucha*** Juz. & Bukasov<br>2n = 3x	Group Chaucha<br>2n = 3x	CHA	CIP 707129
	S.tuberosum L.			
S. tuberosum Andigenum group<br>2n = 4x	*S. tuberosum* subsp. *andigena* Hawkes<br>2n = 4x	Group Andigena<br>2n = 4x	ADG1<br>ADG2	CIP 700921<br>CIP 702853
Chilean cultivated potato species:				
S. tuberosum<br>Chilotaum group<br>2n = 4x	*S. tuberosum* subsp. *tuberosum*<br>2n = 4x	Group Tuberosum<br>2n = 4x	TBR	CIP 705053
-	-	Group Tuberosum - modern improved cultivars<br>2n = 4x	-	-

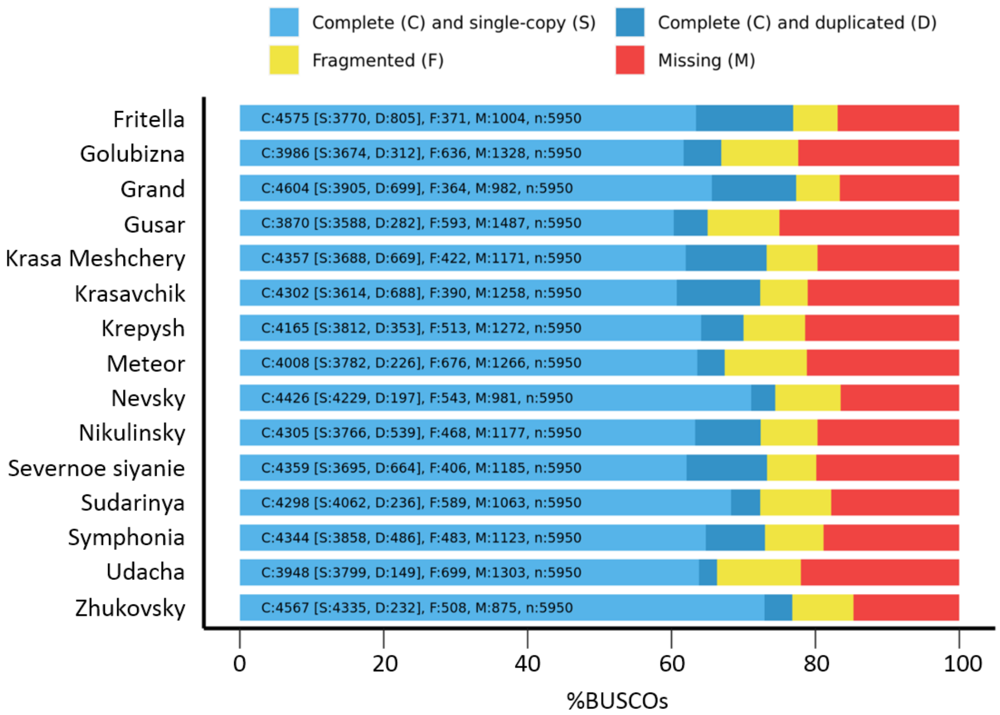

Figure S1. Bar plot with summary assessments for the proportion of BUSCO genes present in 15 genome assemblies of potato cultivars grown in Russia. X-axis: the fraction of BUSCO proteins in the Solanales dataset identified in potato genomes. Y-axis: cultivars. Summary statistics presented at each cultivar bar (n: 5950 is the number of the BUSCO proteins in the Solanales dataset). Bar color description and statistical parameters designation letter shown above the plot: light blue for complete single-copy proteins, dark blue for complete and duplicated proteins, yellow for fragmented proteins, red for missing proteins.

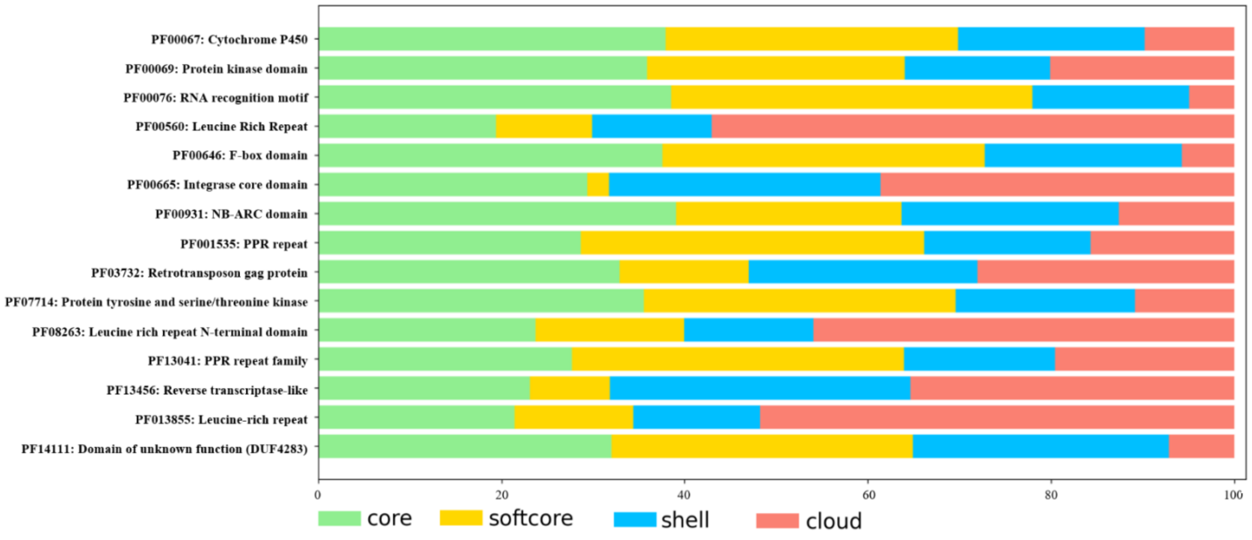

Figure S2. Distribution of the 15 most frequently occurred Pfam domains according to InterproScan annotation in various parts of the pan-genome of the Russian potato cultivars.

	CNL-1	CNL-2	CNL-3	CNL-4	CNL-5	CNL-6	CNL-7	CNL-8	CNL-R	CNL-TNL	n/a
Fritella	9	1	2	0	2	3	7	1	7	5	63
Golubizna	8	2	0	0	2	4	7	1	6	6	63
Grand	10	2	0	0	2	4	8	0	4	6	64
Gusar	7	2	1	1	2	2	10	0	5	6	64
Krasa Meshchery	10	2	1	0	2	4	5	2	5	8	61
Krasavchik	5	3	2	1	4	3	6	1	5	4	64
Krepish	9	2	2	1	1	2	6	1	5	6	64
Meteor	9	2	1	0	3	5	10	1	5	7	58
Nevsky	6	2	0	1	4	6	8	1	6	6	60
Nikulinsky	6	2	2	0	1	3	7	2	7	4	65
Severnoe siyanie	5	3	1	1	4	3	8	1	6	7	63
Sudarinya	10	2	1	0	2	3	8	1	7	6	61
Symphonia	10	2	1	0	2	4	7	1	7	6	60
Udacha	8	2	1	1	3	4	9	0	7	3	61
Zhukovsky	5	3	1	0	2	3	8	1	7	6	66
S.tuberosum	6	5	8	4	12	9	9	9	12	26	0

Figure S3. Fraction of NBS-LRR genes of different classes in genomes of 15 Russian cultivars and in the reference *S. tuberosum* DM1-3 genome (last row). Numbers given in %. Last column shows the fraction of genes not belonging to any class described in DM1-3 genome.

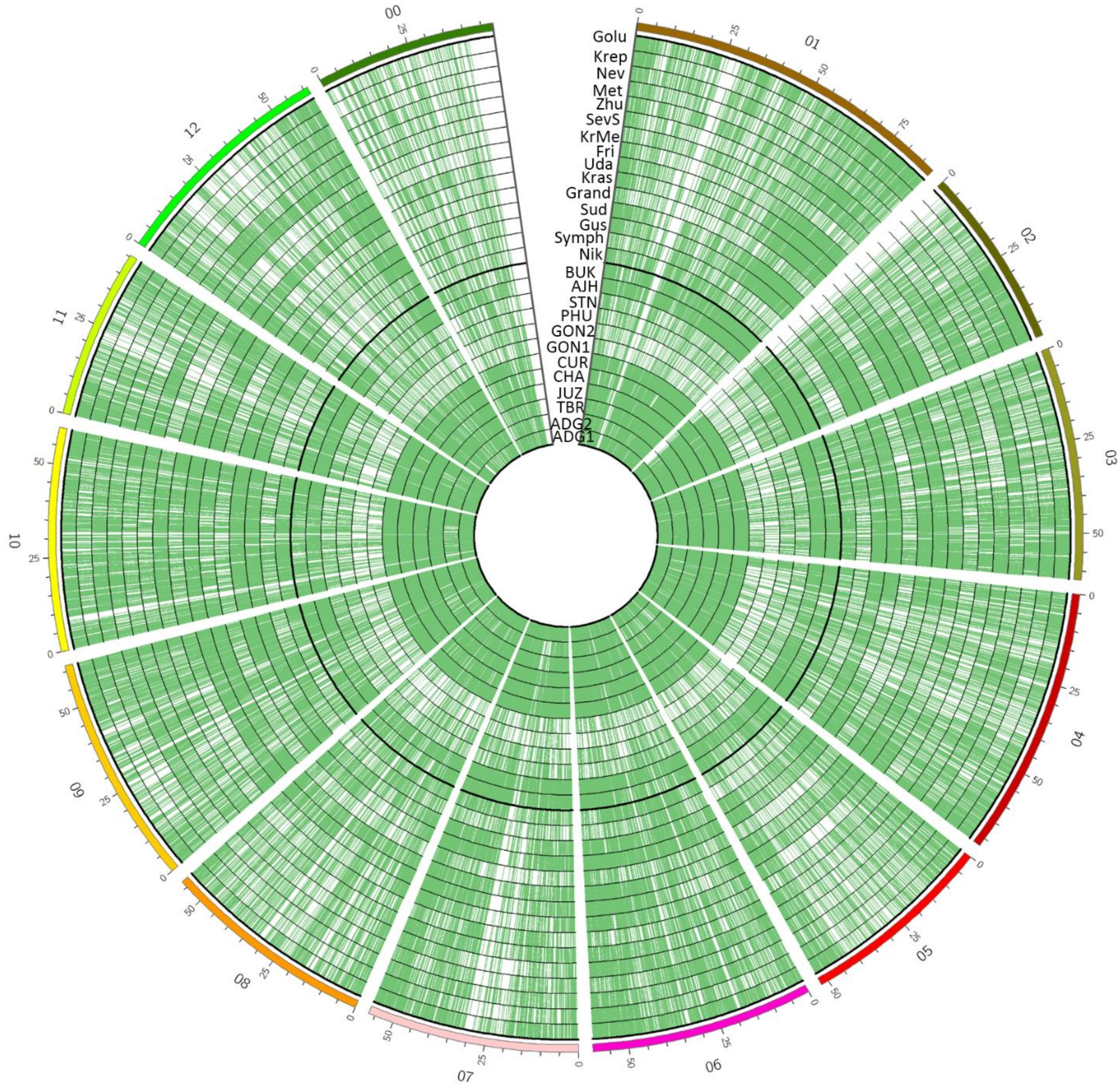

Figure S4. Circos plot for CNV duplications distribution in *S.tuberosum* DM1-3 genome for 15 Russian potato cultivars and 12 South American potato landraces. Sectors correspond to chromosomes, tickmarks correspond to 5 Mb. Green bars correspond to positions of significant CNVs.

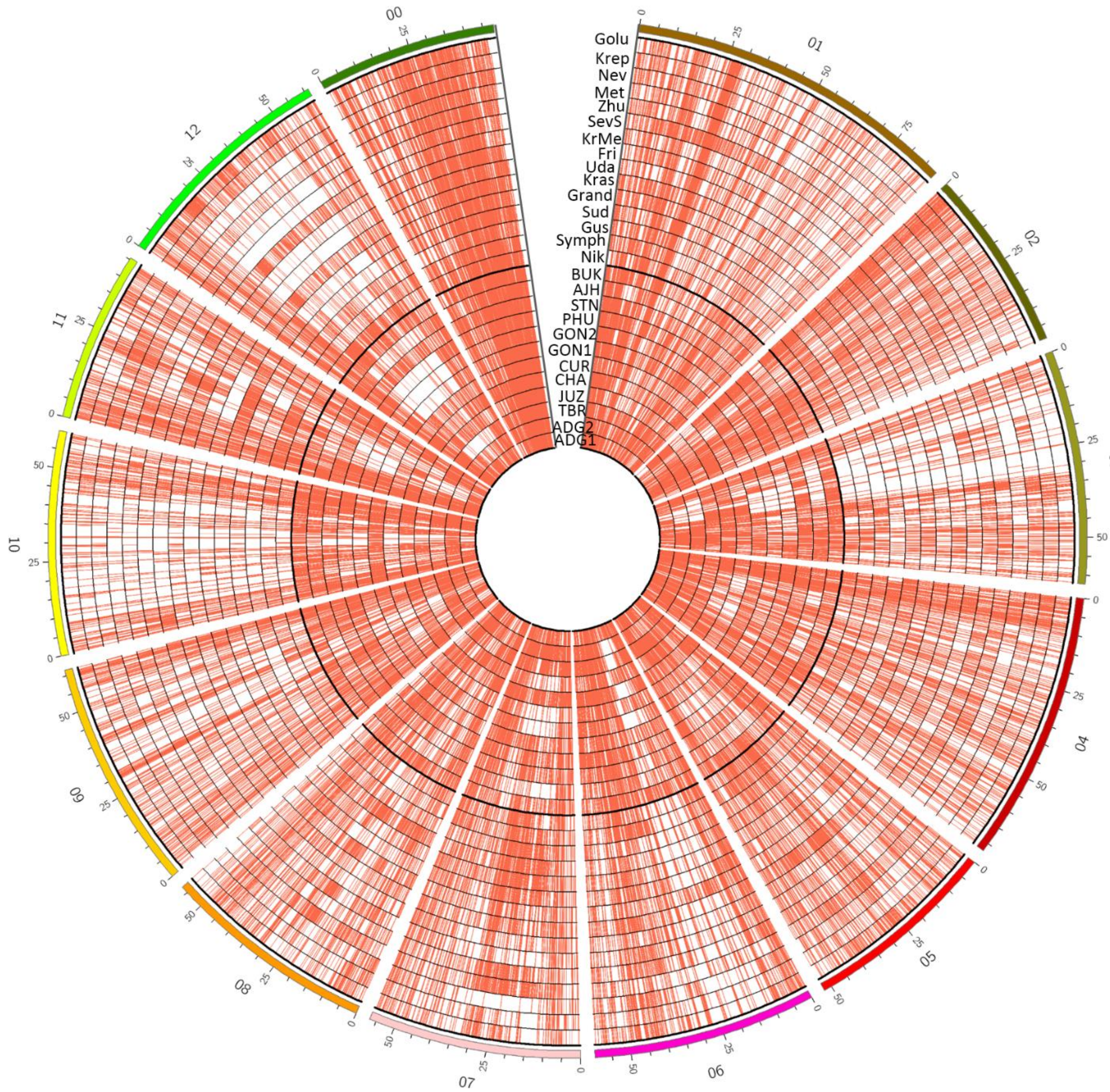

Figure S5. Circos plot for CNV deletions distribution in *S.tuberosum* DM1-3 genome for 15 Russian potato cultivars and 12 South American potato landraces. Sectors correspond to chromosomes, tickmarks correspond to 5 Mb. Sectors correspond to chromosomes. Red bars correspond to positions of significant CNVs.

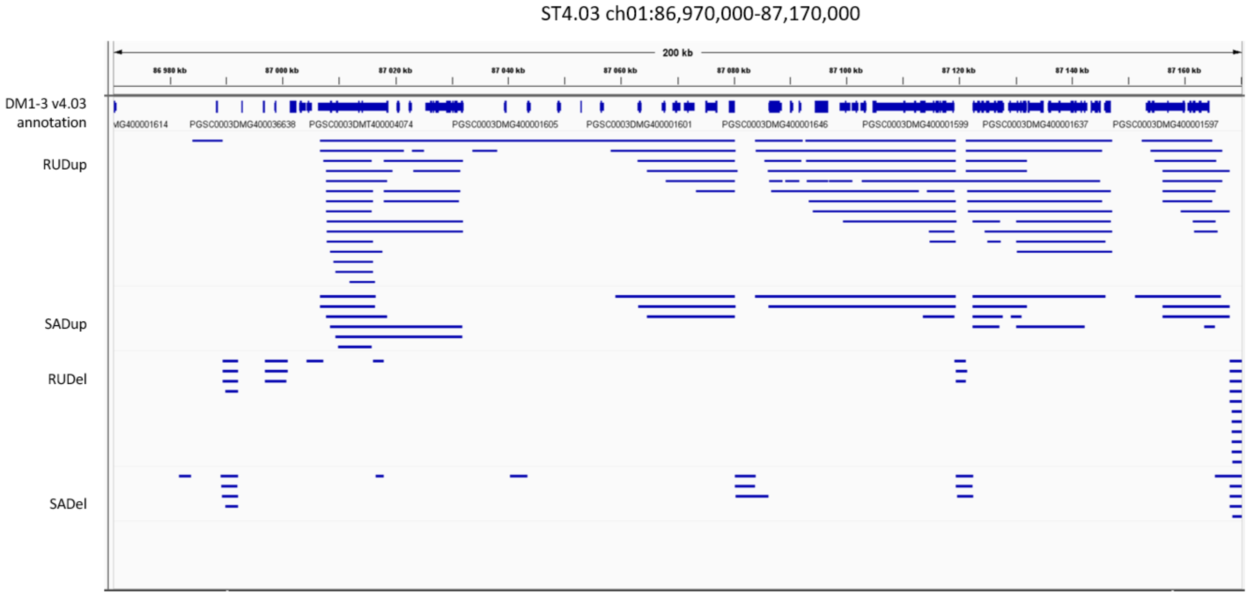

Figure S6. Visualization of the CNVs in Russian potato cultivars and South American potato landraces within the SAUR gene cluster in chromosome 1. Tracks from the top to the bottom: DM1-3 v.4.03 genes, duplications in Russian cultivars (RUDup), duplications in South American accessions (SADup), deletions in Russian cultivars (RUDel), deletions in South American accessions (SADel). Gene locations and the ruler are shown above. The IGV browser was used for visualization.

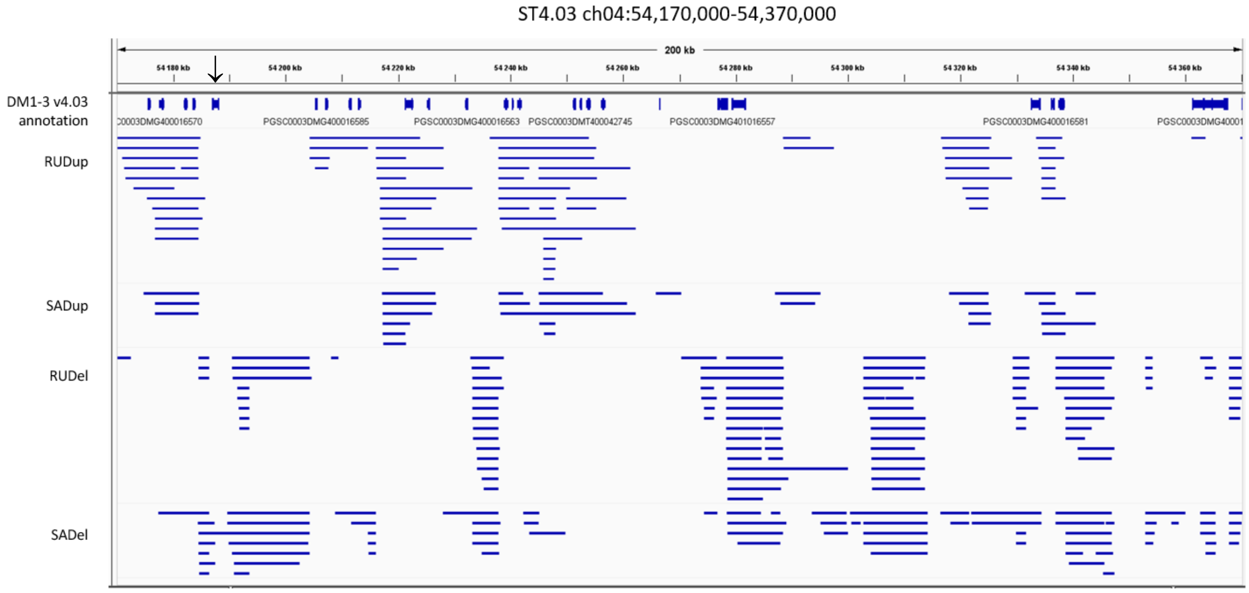

Figure S7. Visualization of the CNVs in Russian potato cultivars and South American potato landraces within the SAUR gene cluster in chromosome 4. Tracks from the top to the bottom: DM1-3 v.4.03 genes, duplications in Russian cultivars (RUDup), duplications in South American accessions (SADup), deletions in Russian cultivars (RUDel), deletions in South American accessions (SADel). Gene locations and the ruler are shown above. Auxin-induced SAUR gene (PGSC0003DMG400016568) is marked by an arrow.

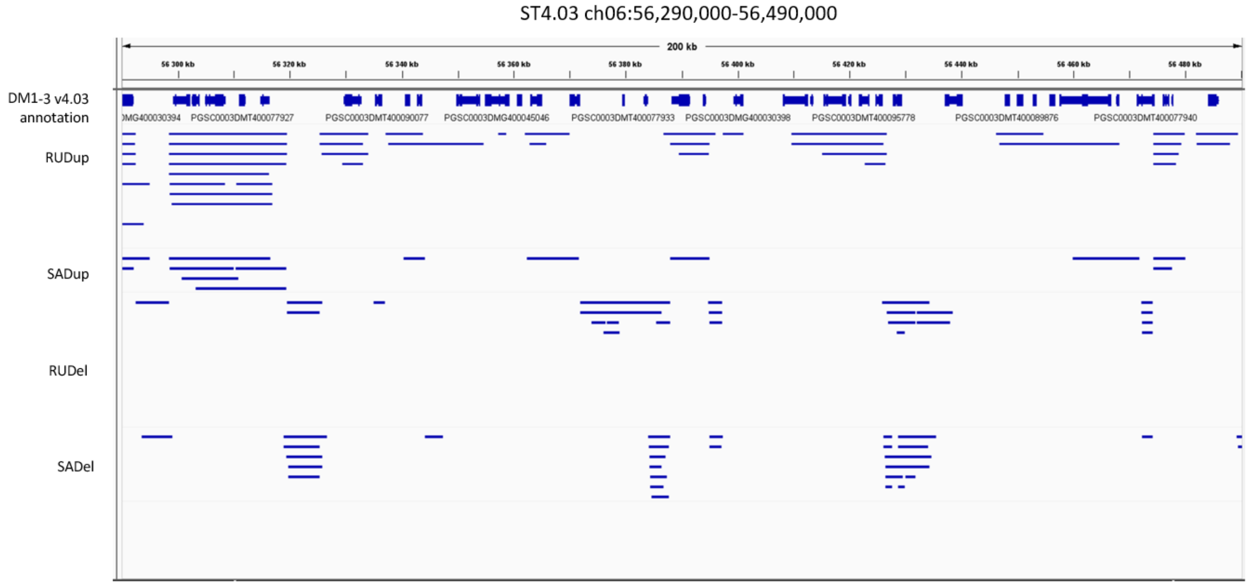

Figure S8. Visualization of the CNVs in Russian potato cultivars and South American potato landraces within the SAUR gene cluster in chromosome 6. Tracks from the top to the bottom: DM1-3 v.4.03 genes, duplications in Russian cultivars (RUDup), duplications in South American accessions (SADup), deletions in Russian cultivars (RUDel), deletions in South American accessions (SADel). Gene locations and the ruler are shown above.

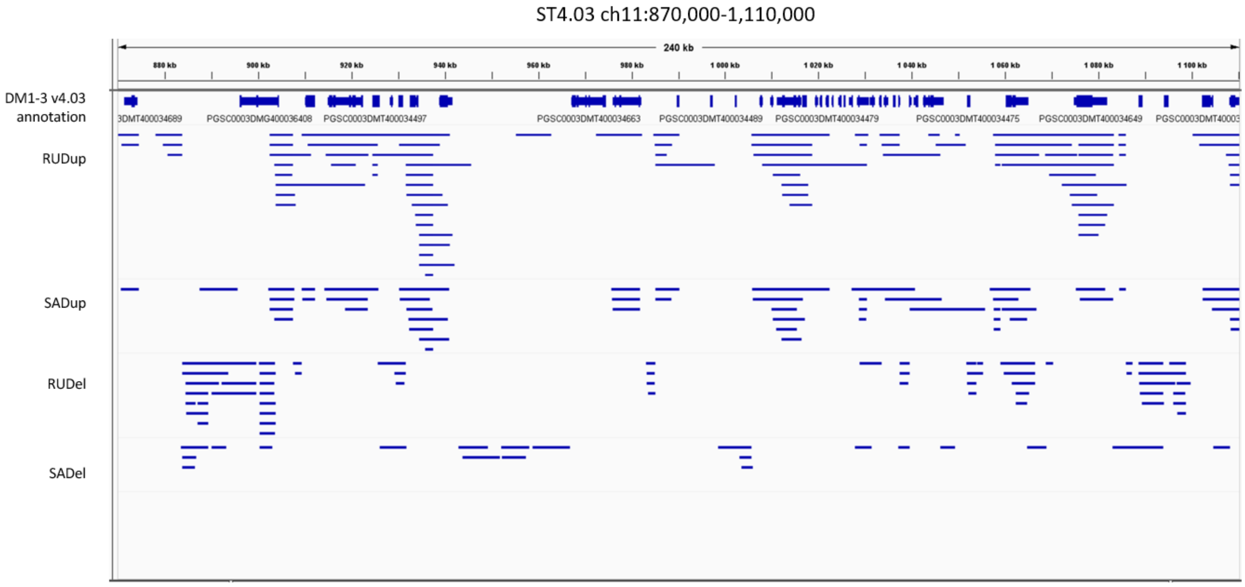

Figure S9. Visualization of the CNVs in Russian potato cultivars and South American potato landraces within the SAUR gene cluster in chromosome 11. Tracks from the top to the bottom: DM1-3 v.4.03 genes, duplications in Russian cultivars (RUDup), duplications in South American accessions (SADup), deletions in Russian cultivars (RUDel), deletions in South American accessions (SADel). Gene locations and the ruler are shown above.

Literature

1. Dodds, K. S. 1962. Classification of cultivated potatoes. In: D. S. Correll (ed.), The potato and its wild relatives. Contributions from Texas Research Foundation, Botanical Studies **4**, 517–539.
2. Hawkes JG (1990) The potato: evolution, biodiversity and genetic resources. Belhaven Press, London
3. Kyriakidou, M.; Achakkagari, S.R.; Gálvez López, J.H.; Zhu, X.; Tang, C.Y.; Tai, H.H.; Anglin, N.L.; Ellis, D.; Strömvik, M.V. Structural Genome Analysis in Cultivated Potato Taxa. *Theor Appl Genet* **2020**, *133*, 951–966, doi:10.1007/s00122-019-03519-6.
4. Ovchinnikova, A.; Krylova, E.; Gavrilenko, T.; Smekalova, T.; Zhuk, M.; Knapp, S.; Spooner, D.M. Taxonomy of Cultivated Potatoes (Solanum Section Petota: Solanaceae): CULTIVATED POTATO TAXONOMY. *Botanical Journal of the Linnean Society* **2011**, *165*, 107–155, doi:10.1111/j.1095-8339.2010.01107.x.
5. Spooner, D.M.; Ghislain, M.; Simon, R.; Jansky, S.H.; Gavrilenko, T. Systematics, Diversity, Genetics, and Evolution of Wild and Cultivated Potatoes. Bot. Rev. **2014**, 80, 283–383, doi:10.1007/s12229-014-9146-y.
6. Spooner, D.M.; Núñez, J.; Trujillo, G.; del Rosario Herrera, M.; Guzmán, F.; Ghislain, M. Extensive Simple Sequence Repeat Genotyping of Potato Landraces Supports a Major Reevaluation of Their Gene Pool Structure and Classification. Proc. Natl. Acad. Sci. U.S.A. **2007**, 104, 19398–19403, doi:10.1073/pnas.0709796104.
